# *HANABA TARANU (HAN)* Bridges Meristem and Organ Primordia Boundaries through *PINHEAD*, *JAGGED*, *BLADE-ON-PETIOLE2* and *CYTOKININ OXIDASE 3* during Flower Development in *Arabidopsis*

**DOI:** 10.1371/journal.pgen.1005479

**Published:** 2015-09-21

**Authors:** Lian Ding, Shuangshuang Yan, Li Jiang, Wensheng Zhao, Kang Ning, Jianyu Zhao, Xiaofeng Liu, Juan Zhang, Qian Wang, Xiaolan Zhang

**Affiliations:** Department of Vegetable Sciences, Beijing Key Laboratory of Growth and Developmental Regulation for Protected Vegetable Crops, China Agricultural University, Beijing, China; Peking University, CHINA

## Abstract

Shoot organ primordia are initiated from the shoot apical meristem and develop into leaves during the vegetative stage, and into flowers during the reproductive phase. Between the meristem and the newly formed organ primordia, a boundary with specialized cells is formed that separates meristematic activity from determinate organ growth. Despite interactions that have been found between boundary regulators with genes controlling meristem maintenance or primordial development, most boundary studies were performed during embryogenesis or vegetative growth, hence little is known about whether and how boundaries communicate with meristem and organ primordia during the reproductive stage. We combined genetic, molecular and biochemical tools to explore interactions between the boundary gene *HANABA TARANU* (*HAN*) and two meristem regulators *BREVIPEDICELLUS (BP)* and *PINHEAD* (*PNH*), and three primordia-specific genes *PETAL LOSS (PTL)*, *JAGGED* (*JAG*) and *BLADE-ON-PETIOLE (BOP)* during flower development. We demonstrated the key role of *HAN* in determining petal number, as part of a set of complex genetic interactions. *HAN* and *PNH* transcriptionally promote each other, and biochemically interact to regulate meristem organization. HAN physically interacts with JAG, and directly stimulates the expression of *JAG* and *BOP2* to regulate floral organ development. Further, *HAN* directly binds to the promoter and intron of *CYTOKININ OXIDASE 3* (*CKX3*) to modulate cytokinin homeostasis in the boundary. Our data suggest that boundary-expressing HAN communicates with the meristem through the PNH, regulates floral organ development via JAG and BOP2, and maintains boundary morphology through CKX3 during flower development in *Arabidopsis*.

## Author Summary

The shoot apical meristem is the stem cell pool in plants that gives rise to all above-ground organs including leaves, flowers and fruits. Between the meristem and the newly formed organ primordia, a boundary with specialized cells is formed to separate them. Boundary genes are specifically expressed in boundaries and function in boundary formation and maintenance. Previous studies showed that boundary genes interact with meristem regulators and primordia genes during embryogenesis or leaf development. But whether and how boundaries communicate with meristem and organ primordia during flower development remains largely unknown. Here we combined genetic, molecular and biochemical tools to explore interactions between the boundary gene *HANABA TARANU* (*HAN*) and two meristem regulators *BREVIPEDICELLUS (BP)* and *PINHEAD* (*PNH*), and three primordia-specific genes *PETAL LOSS (PTL)*, *JAGGED* (*JAG*) and *BLADE-ON-PETIOLE (BOP)* during flower development. We showed that boundary-expressing HAN communicates with the meristem through PNH, regulates floral organ development via *JAG* and *BOP2*, and maintains boundary morphology through *CYTOKININ OXIDASE 3* (*CKX3*)-mediated cytokinin homeostasis. Thus, our findings shed light on the “bridge” role of boundaries between meristem and organ primordia during flower development in *Arabidopsis*.

## Introduction

Leaves and flowers originate from the shoot apical meristem (SAM), which contains pluripotent stem cells and resides at the tip of each stem. Primordia are initiated from the peripheral zone of the SAM in a predictable pattern, and develop into leaves during the vegetative stage, and into flowers during the reproductive phase. Each flower consists of four concentric whorls of organ types: the protective sepals, the showy petals, the male stamens, and the female carpels [1]. Between the meristem and newly formed leaf or flower primordia, a boundary forms with specialized cells that separate meristematic activity from determinate organ growth [2]. Cells in the boundary have reduced rates of cell division, concave surfaces, elongated shapes, and exhibit low auxin concentration compared to the adjacent cells in meristems or primordia [3–6]. There are two types of boundaries in the developing shoot apices. M-O (meristem-organ) boundaries separate leaf and flower primordia from the SAM, whereas O-O (organ-organ) boundaries develop between individual floral organs and create space between them [2, 7].

Based on boundary-specific expression patterns and mutant defects in boundary formation, organ separation, SAM initiation and maintenance, branching, or floral organ patterning, several transcription factors have been identified as important boundary regulators, including *CUP-SHAPED COTYLEDONS 1*, *2* and *3 (CUC1*, *CUC2*, *CUC3)*, *LATERAL SUPPRESSOR (LAS)*, *LATERAL ORGAN BOUNDARIES (LOB)*, *JAGGED LATERAL ORGANS (JLO)*, *LATERAL ORGAN FUSION (LOF)*, *HANABA TARANU* (*HAN*), *SUPERMAN (SUP)* and *RABBIT EARS (RBE)*[5, 8–21]. Interactions have been found between boundary regulators and genes controlling meristem maintenance or primordia development. For example, *CUC* genes promote SAM formation via the activation of meristem marker *SHOOT MERISTEMLESS* (*STM*), and in return, *STM* represses *CUC* expression in the meristem [9, 22]. *CUC* genes are also inhibited by primordia marker *ASYMMETRIC LEAVES 1 (AS1)* and *AS2* in the organ primordia [23–25]. However, as most boundary studies were performed during embryogenesis or vegetative growth, little is known about how boundary regulators communicate with meristem and organ primordia during the reproductive stage.

The boundary regulator *HAN* encodes a GATA-3 type transcription factor with a single zinc finger domain and plays a role in *Arabidopsis* flower development. *HAN* is expressed at the boundaries between meristem and floral organ primordia and at the boundaries of floral organs [13]. Mutation of *HAN* leads to fused sepals, and reduced numbers of petals and stamens [13]. The meristem regulator *KNAT1 /BREVIPEDICELLUS (BP)* encodes a KNOTTED1-LIKE HOMEOBOX (KNOX) class I homeobox gene that is required for inflorescence architecture. Disruption of *BP* function results in short internodes and pedicels, and downward-oriented siliques [26, 27]. Similarly, *ARGONAUTE* 10/*PINHEAD* (*PNH*), a founding member of the *ARGONAUTE* family, is a regulator of meristem maintenance that acts by sequestering miR166/165, preventing its incorporation into an *ARGONAUTE 1* complex [28–31]. In *pnh* mutants, phenotypes are pleiotropic including an SAM occupied by pin-like structures, increased numbers of floral organs, and disrupted embryo and ovule development [32]. In primordia, indeterminate meristematic activities are repressed and primordia-specific genes are induced to ensure proper determinate organ development [2, 23, 24]. *PETAL LOSS (PTL)*, *JAGGED* (*JAG*) and *BLADE-ON-PETIOLE (BOP)* belong to the class of primordia-specific genes that regulates flower organ development [32]. *PTL* is expressed in the margins of developing sepals, petals and stamens, and ensures normal petal initiation by maintaining auxin homeostasis [33, 34]. Loss of function of *PTL* leads to reduced numbers of petals and disrupted petal orientation [11, 35, 36]. *JAG*, a putative C2H2 zinc finger transcription factor, expresses in the initiating primordia but not the meristem, and regulates lateral organ development in *Arabidopsis* [37]. A *JAG* knockout mutant displays serrated sepals and narrow petals [37, 38]. *JAG* controls cell proliferation during organ growth by maintaining tissues in an actively dividing state [37], and acts redundantly with *NUBBIN*, a *JAGGED*-like gene, to control the shape and size of lateral organs [39]. *BOP1*/2 specify BTB/POZ domain proteins and express in the base of flower primordia. They function redundantly to control flower and leaf development [17, 40–42]. Loss of function of *BOP1* and *BOP2* results in increased petal numbers, lack of floral organ abscission and leafy petioles [42–44].

Whether and how boundary genes interact with meristem-related regulators and primordia-specific genes during flower development remains largely unknown. In this study, we combined genetic, molecular and biochemical tools to explore interactions between the boundary gene *HAN* and two meristem regulators *(BP* and *PNH)*, and three primordia-specific genes *(PTL*, *JAG* and *BOP1/2)* that function in flower development. We found that *HAN* plays a central role among these seven regulators in the control of petal development. At the transcriptional level, *HAN* promotes *PNH* transcription and represses *BP* expression, *BP* represses *PNH* while *PNH* positively feeds back on the expression of *HAN*. At the protein level, HAN physically interacts with PNH and PNH interacts with BP to regulate meristem organization. HAN also interacts with JAG, and directly promotes the expression of *JAG* and *BOP2* to regulate floral organ development. Further, HAN directly stimulates *CYTOKININ OXIDASE 3* (*CKX3*) expression to modulate cytokinin levels in the boundary. Therefore, our data suggest a new link by which *HAN* communicates with the meristem through *PNH*, regulates primordia development via *JAG* and *BOP2*, and maintains boundary morphology through CKX3-mediated cytokinin homeostasis during flower development in *Arabidopsis*.

## Results

### Genetic interactions of *HAN* with meristem- and primordial-regulators during flower development in *Arabidopsis*

**Fig 1:**
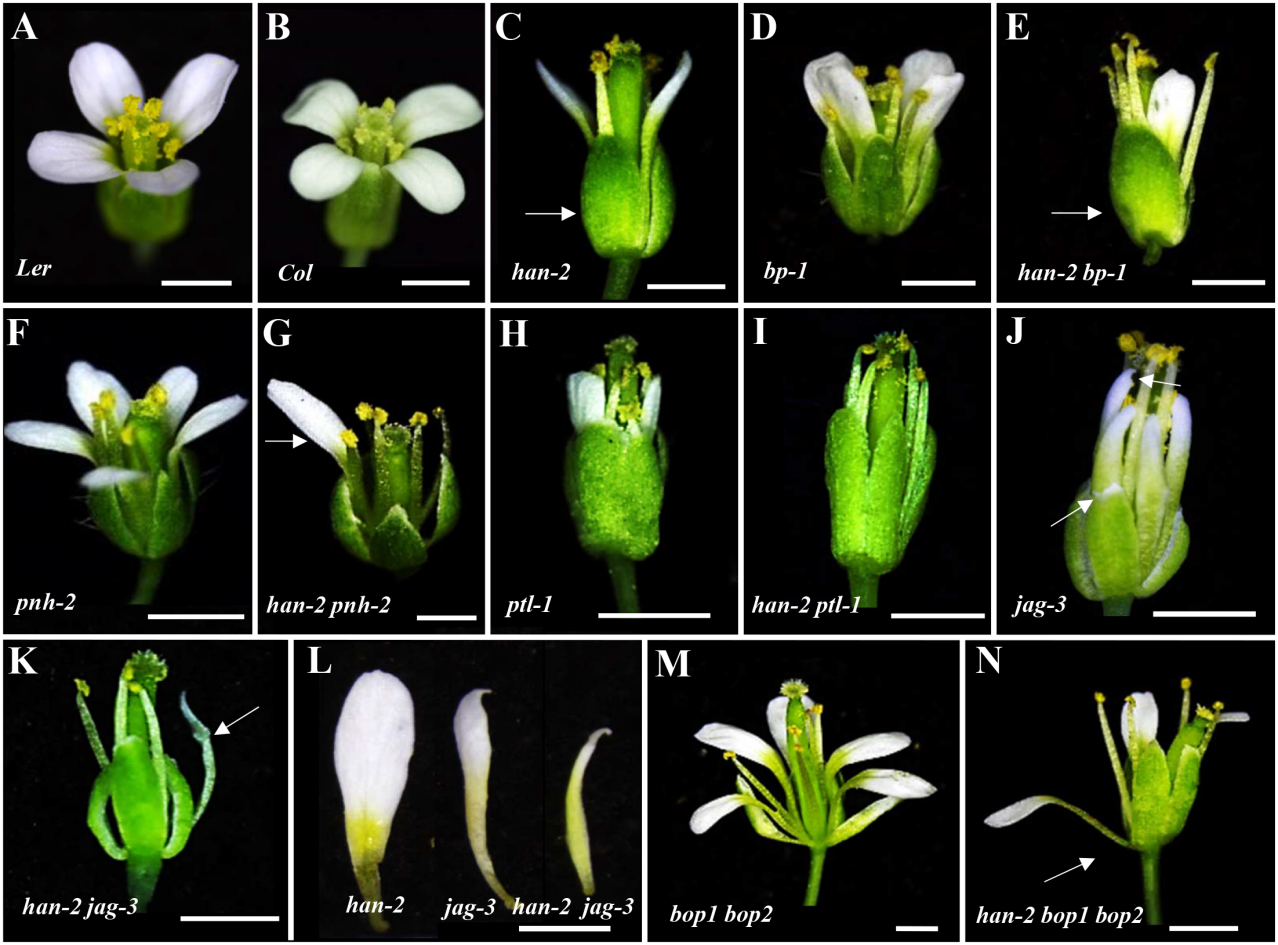
Genetic interactions of *HAN* with meristem and primordia regulators during flower development. (A-B) A Landsberg erecta flower (A) and a Columbia flower (B). (C) Representative image of *han-2* single mutant with fused sepals (arrow) and reduced petals and stamens. (D-E) flowers in *bp-1* (D) and *han-2 bp-1* double mutant (E) with fused sepals (arrow) and reduced petals. (F-G) Floral phenotypes of *pnh-2* mutant (F) and *han-2 pnh-2* double mutant (G) showed a significantly reduced number of petals (arrow). (H-I) Representative flowers of *ptl-1 (Col)* (H) and *han-2 ptl-1* double mutants (I) with loss of petals. (J) Images of *jag-3* flower with serrate sepals and petals (arrows). (K) *han-2 jag-3* with reduced number of petals. (L) The petals were narrower in the *jag-3 han-2* double mutant. (M, N) Floral phenotypes of *bop1 bop2* (M) and *han-2 bop1 bop2* triple mutant (N) showed that petal number was rescued. Arrow in (N) indicates a petal in the first whorl. Bars = 1mm.

**Fig 2:**
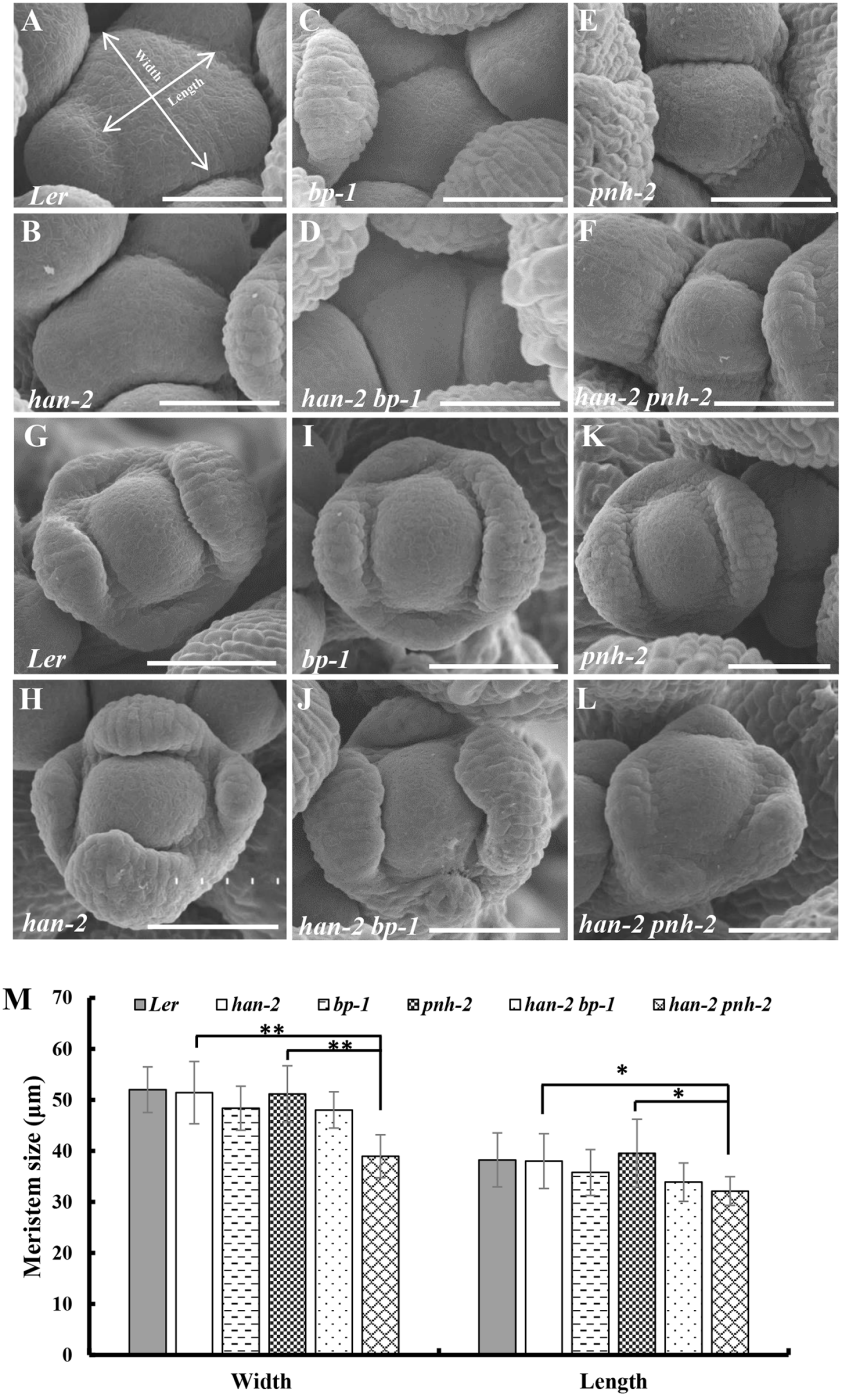
Scanning electron micrographs of the inflorescence meristems and flowers. (A-F) Inflorescence meristems of wild-type *Ler* (A), *han-2* (B), *bp-1* (C), *han-2 bp-1* (D), *pnh-2* (E), *han-2 pnh-2* (F) in 40-day-old plants. (G-L) Flowers of wild-type *Ler* (G), *han-2* (H), *bp-1* (I), *han-2 bp-1* (J), *pnh-2* (K), *han-2 pnh-2* (L). (M) Statistical analyses of inflorescence meristems size from wild-type *Ler*, *han-2*, *bp-1*, *pnh-2*, *han-2 bp-1*, *han-2 pnh-2*. The measuring method was showed in Fig 2A. Values are the means of 6–10 plants grown under the same condition. Asterisks and double asterisks indicate that the values in the mutants were significantly different from the wild type at P < 0.05 and P < 0.01, respectively (unpaired t test, P > 0.05). Note that the inflorescence meristem and floral meristem of *han-2 pnh-2* is smaller and taller than the single mutant *han-2* and *pnh-2*. Bars = 40 μm.

Mutation in *HAN* results in reduced numbers of petals and stamens, and fused sepals [13]. In contrast to the wild-type flower with four sepals and four petals, the *han-2* mutant has an average of only 3.4 sepals and 2.6 petals in the *Ler* or *Col* background (Fig 1A–1C, Table 1). In order to explore the potential genetic interactions between *HAN*, meristem regulators, and primordia-specific genes during flower development, we generated double or triple mutant combinations of *han-2* with *bp-1*, *pnh-2*, *ptl-1*, *jag-3* and *bop1 bop2* (Fig 1 and S1 Fig). Firstly, we explored the genetic interaction of *HAN* with meristem regulator *BP*. The *bp-1* mutant shows a normal number of floral organs, with downward-pointing flowers and a compact inflorescence (Fig 1D and S1B Fig) [26]. The number of petals and sepals was reduced in a *han-2 bp-1* double mutant, with an average of 1.7±0.1 (n = 120) petals (Fig 1E and S1C Fig, Table 1). The phenotype of fused sepals is similar to *han-2*. Then, we examined the genetic interaction of *HAN* with *PNH*, whose mutations result in increased numbers of petals. The average petal number was 4.3±0.1 (n = 40) in the *pnh-2* mutant (Fig 1F, Table 1). *han-2 pnh-2* double mutants have fewer petals than the *han-2* single mutant (Fig 1G and S1D–S1G Fig, Table 1). Therefore, mutation of meristem regulators *BP* and *PNH* enhanced the petal loss phenotype of *han-2*. Given that both BP and PNH are meristem regulators [24, 45], we next explored the phenotypes of meristem organization upon induction of *han-2* into *bp-1* or *pnh-2* mutant background (Fig 2). In inflorescence meristems (IM) and floral meristems (FM), no obvious changes were observed in the meristem organization of the single mutant *han-2* and *bp-1*, or double mutant *han-2 bp-1* as compared to the wild-type (Fig 2A–2D, 2G–2J, and 2M). However, mutation of *HAN* greatly enhanced the smaller and taller IM and FM phenotype in the *pnh-2* (Fig 2E and 2F, 2K and 2L, and 2M), suggesting that *HAN* and *PNH* coordinatively regulate meristem organization in *Arabidopsis*.

**Table 1:** Characterization of the number of floral organs in different mutant lines.

Mutant lines		Sepals	Petals	Stamens	Carpels
*Ler*	n = 40	4.0±0.0	4.0±0.0	5.5±0.1	2.0±0.0
*han-2(Ler)*	n = 40	3.4±0.1	2.6±0.2	4.1±0.1	2.0±0.0
*jag-3(Ler)*	n = 40	3.8±0.1	3.8±0.1	5.6±0.1	2.0±0.0
*han-2 jag-3(Ler)*	n = 40	3.6±0.1	1.8±0.2^ab^	4.0±0.2^b^	2.0±0.0
*pnh-2(Ler)*	n = 40	4.1±0.0	4.3±0.1	5.4±0.1	2.8±0.1
*han-2 pnh-2(Ler)*	n = 40	4.2±0.2^a^	1.7±0.2^ab^	4.5±0.2^Ab^	2.4±0.1^a^
*bp-1(Ler)*	n = 120	4.0±0.0	4.0±0.0	5.5±0.1	2.0±0.0
*bp-1 han-2(Ler)*	n = 120	2.5±0.1^ab^	1.7±0.1^ab^	3.9±0.1^b^	2.0±0.0
*Col*	n = 40	4.0±0.0	4.0±0.0	5.8±0.1	2.0±0.0
*han-2(Col)*	n = 60	3.4±0.1	2.6±0.1	4.3±0.1	2.0±0.0
*ptl-1(Col)*	n = 60	3.8±0.1	1.9±0.2	5.9±0.0	2.0±0.0
*han-2 ptl-1(Col)*	n = 60	4.2±0.1^ab^	0.9±0.2^ab^	5.1±0.1^ab^	2.0±0.0
*bop1 bop2(Col)*	n = 70	4.0±0.1	5.2±0.1	5.4±0.1	2.0±0.0
*han-2 bop1 bop2(Col)*	n = 70	3.9±0.0^a^	3.6±0.1^ab^	4.2±0.1^b^	2.0±0.0

^A^ and ^a^ represents P-value ≦ 0.05 and P-value ≦ 0.01 as compared to that in *han-2*, respectively.

^b^ means P-value ≦ 0.01 as compared with to its corresponding single or double mutants other than han-2.

Next, we examined the genetic interactions of *HAN* with the primordial- specific genes *PTL*, *JAG* and *BOP1/2* during flower development. The single mutant *ptl-1* displays defective flowers with reduced petal numbers and disrupted petal orientation (Fig 1H and S1J Fig) [11, 35], and introduction of *han-2* into the *ptl-1* mutant further decreased petal numbers (Fig 1I and S1H–S1K Fig), resulting in an average of 0.9±0.2 petals (n = 60) in each *han-2 ptl-1* double mutant flower (Table 1). *JAG* is required for lateral organ morphology, and loss of function of *JAG* results in flowers with narrow floral organs, jagged organ margins and slightly reduced numbers of petals (Fig 1J and S1L Fig, Table 1) [37, 38]. Loss of function of both *HAN* and *JAG* genes led to reduced petal numbers (Fig 1K and S1L and S1M Fig). The average number of petals in a *han-2 jag-3* double mutant was 1.8±0.2 (n = 40), a more severe phenotype than that in the *han-2* single mutants (Table 1). Similarly, the sepals were more serrated and the petals were narrower in *han-2 jag-3* than in a *jag-3* single mutant (Fig 1L and S1M Fig), suggesting that *HAN* and *JAG* have a synergistic effect on regulation of petal number, and sepal and petal morphology. In the *han-2 bop1 bop2* triple mutant, on the other hand, the number of petals was largely rescued to normal (3.6±0.1) compared to 5.2±0.1 in *bop1 bop2* mutants (Fig 1M and 1N and S1N and S1O Fig, Table 1). However, there were two developmental phenotypes in the *han-2 bop1 bop2* triple mutants similar to the phenotype of *bop1 bop2* mutants: 1) petaloid tissue replacing the sepal (Fig 1M and 1N); and 2) floral organs never fall off due to lack of an abscission zone (S2A and S2B Fig). However, the ectopic leaf tissues on the petioles observed in *bop1 bop2* double mutants were mostly rescued upon introduction of *han-2* (S2C Fig).

### Transcriptional communications between boundary, meristem and floral organs

**Fig 3:**
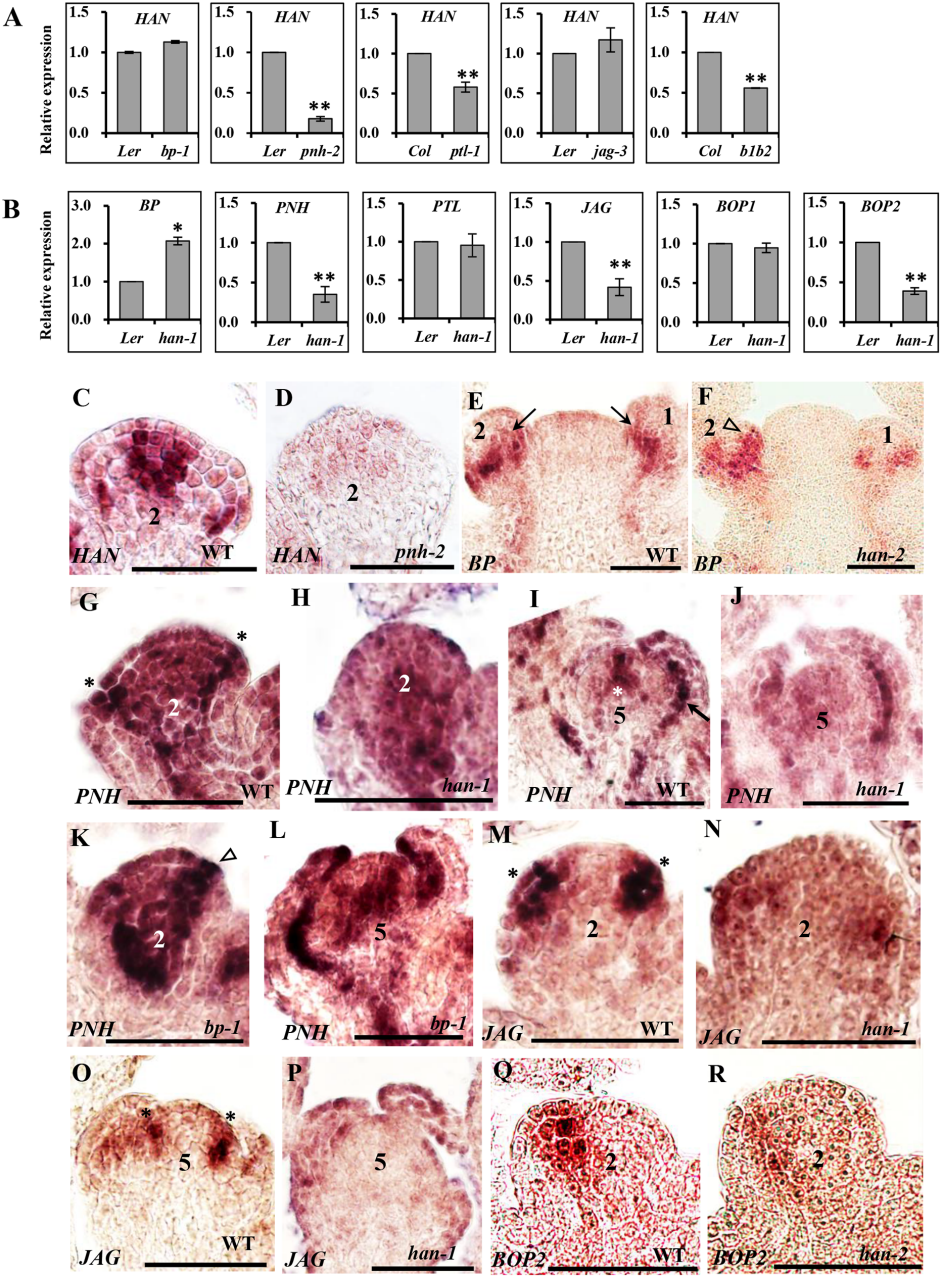
Transcriptional analyses by quantitative real time RT-PCR and *in situ* hybridization in different mutant backgrounds. (A-B) qRT-PCR analyses of *HAN* in the inflorescence of different mutant lines (A) and transcript levels of *BP*, *PNH*, *PTL*, *JAG*, *BOP1* and *BOP2* in the *han-1* mutant (B). *Arabidopsis ACTIN2* was used as an internal standard to normalize the templates. Three biological replicates were performed for each gene, and the bars represent the standard deviation. Asterisks and double asterisks indicate that expression levels in the mutants were significantly different from the wild type at P < 0.05 and P < 0.01, respectively. Lack of asterisk indicates the difference was not significant (unpaired t test, P > 0.05). (C-R) *in situ* hybridization. (C-D) *HAN* expression in wild-type (C) and *pnh-2* (D) mutant flowers. (E-F) *BP* is expressed in the base of flower primordia (arrows) in wild-type (E), while *BP* signal appears to expand to include the initiating sepal primordia (triangle) in the *han-2* mutant (F). (G-L) *PNH* expression in wild-type (G, I), *han-1* (H, J) and *bp-1* (K, L). In wild-type, *PNH* signal is detected in the floral meristem, the adaxial side of sepal primordia (asterisks in G), and the provascular tissue (arrow in I). In the *han-1* mutant, *PNH* signal is greatly decreased (H, J), while in the *bp-1* mutant, *PNH* expression is substantially increased (K, L). (M-P) *JAG* mRNA is located in developing organ primordia (asterisks) (M, O), and is reduced in the *han-1* mutant (N, P); (Q-R) RNA localization of *BOP2* in wild-type (Q) and *han-2* mutant (R). In wild-type, *BOP2* is predominantly expressed in the boundary between FM and sepal primordia at stage 2, and the expression of *BOP2* is decreased in *han-2* mutant plants (R). Seven biological samples were used for each probe. Numbers over each section represent the stage of floral development [1, 70]. The same probe concentration was used in the mutant and wild-type inflorescences and the slides were developed for the same period of time. Bars = 50μm.

The mutant phenotypes suggested that *HAN*, together with *BP*, *PNH*, *PTL*, *JAG* and *BOP1/2* regulates flower development via complex genetic interactions. To explore this potential regulatory network at the transcriptional level, gene expression was quantified by real time qRT-PCR in the mutant lines (Fig 3A and 3B and S3 Fig), and temporal and spatial expression patterns of these regulators were further analyzed by *in situ* hybridization (Fig 3C–3R and S4 Fig). *HAN* transcripts localize to the boundaries between the meristem and developing organ primordia, the junctional domain between the SAM and the stem, and the boundaries between different floral whorls [13]. qRT-PCR showed that the expression level of *HAN* was significantly reduced in the *pnh-2*, *ptl-*1 and *bop1 bop2* mutant inflorescences, especially in the *pnh-2* mutant, where transcript accumulation of *HAN* was decreased to 17% of the wild-type level, while there was no significant change in the *jag-3* or *bp-1* mutant plants (Fig 3A), suggesting that *PNH*, *PTL* and *BOP1/2* promote *HAN* expression. Consistently, *in situ* hybridization showed that the *HAN* signal was dramatically decreased and diffused in the *pnh-2* mutant (Fig 3C and 3D and S4A and S4B Fig). However, no obvious difference was detected for the signal of *HAN* in the *bop1 bop2*, *ptl-1*, *jag-3* or *bp-1* mutant as compared to that in wild-type (WT) (S4C–S4H Fig), probably due to such levels of reduction in the *bop1 bop2* (-1.7 fold) and *ptl-1*(-1.8 fold) are visibly undetectable by *in situ* hybridization.

The meristem regulator *BP* is expressed in the cortex of developing pedicels within the base of floral primordia in WT plants (arrows in Fig 3E). In the *han-2* mutant, *BP* signal appeared to expand into the initiating sepal primordia (triangle in Fig 3F), or expand to the abaxial of the sepal primordia in stage 5 (S4I and S4J Fig). qRT-PCR verified that the expression of *BP* was upregulated 2-fold in the *han-1* inflorescence (Fig 3B), suggesting that the boundary gene *HAN* may inhibit the expression of *BP* from expanding into organ primordia. As for the other meristem regulator *PNH*, qRT-PCR showed that *PNH* transcription was reduced nearly 3-fold in *han-1*, but increased 4-fold in *bp-1* (Fig 3B and S3B Fig). Consistently, the mRNA signal of *PNH* was concentrated in the adaxial side of sepal primordia (asterisk in Fig 3G), the floral meristem (FM) (Fig 3G and asterisk in Fig 3I), and the provascular tissue (arrow in Fig 3I) [28]. In the *han-1* mutant, *PNH* signal was decreased, especially at the adaxial side of sepal primordia at stage 2 (Fig 3H), and the center of FM at stage 5 (Fig 3J). In the *bp-1* mutant, *PNH* signal was greatly enhanced (Fig 3K and 3L), supporting the conclusion that *HAN* promotes while *BP* inhibits *PNH* expression during flower development. Therefore, the boundary-expressing *HAN* and the two meristem regulators *PNH* and *BP* form a regulatory feedback loop, in which *HAN* promotes *PNH* and represses *BP* transcription, and *BP* represses *PNH* while *PNH* acts positively on *HAN* expression.

The organ primordia-expressed gene *PTL* appears to be unaffected in the *han* mutant as detected by qRT-PCR (Fig 3B) and *in situ* hybridization (S4K and S4L Fig), and it is expressed in the margins of developing sepals and in the boundary between sepals and sepal primordia as previously reported (S4K and S4L Fig) [11]. In the five tested mutant lines (*han-1*, *bp-1*, *pnh-2*, *ptl-1* and *bop1 bop2*), *JAG* expression was significantly downregulated, with the lowest expression in *bp-1* (Fig 3B and S3D Fig), implying that *JAG* may be a downstream gene in the regulatory network. Consistently, about 25% of the *han-1* mutant flowers almost abolished the *JAG* signal as compared to the enriched mRNA level in the emerging sepal primordia and stamen primordia in WT flowers (asterisks in Fig 3M–3P) [38], supporting the idea that *HAN* can stimulate *JAG* expression in organ primordia. Previous studies showed that *BOP1* and *BOP2* function redundantly and exhibit similar expression patterns [41, 46]. We found the expression of *BOP1* displayed slight changes in all five mutant lines (*han-1*, *bp-1*, *pnh-2*, *ptl-1* and *jag-3*) (Fig 3B and S3E Fig), while the expression of *BOP*2 was significantly repressed in the *han-1* or *jag-3* mutant, and significantly enhanced in the *bp-1* mutant (Fig 3B and S3F Fig). Both *BOP1* and *BOP2* were expressed at the boundary between FM and sepal primordia, and base of sepals and other floral organs as previously reported (Fig 3Q and 3R and S4M–S4P Fig) [41, 47]. In *han* mutant flowers, the *BOP2* signal appeared low (Fig 3R and S4P Fig), but the *BOP1* signal remained unchanged (S4M and S4N Fig), suggesting that transcription of *BOP1* and *BOP2* may be under different regulatory control during flower development.

### Protein interactions between HAN, PNH, BP and JAG

**Fig 4:**
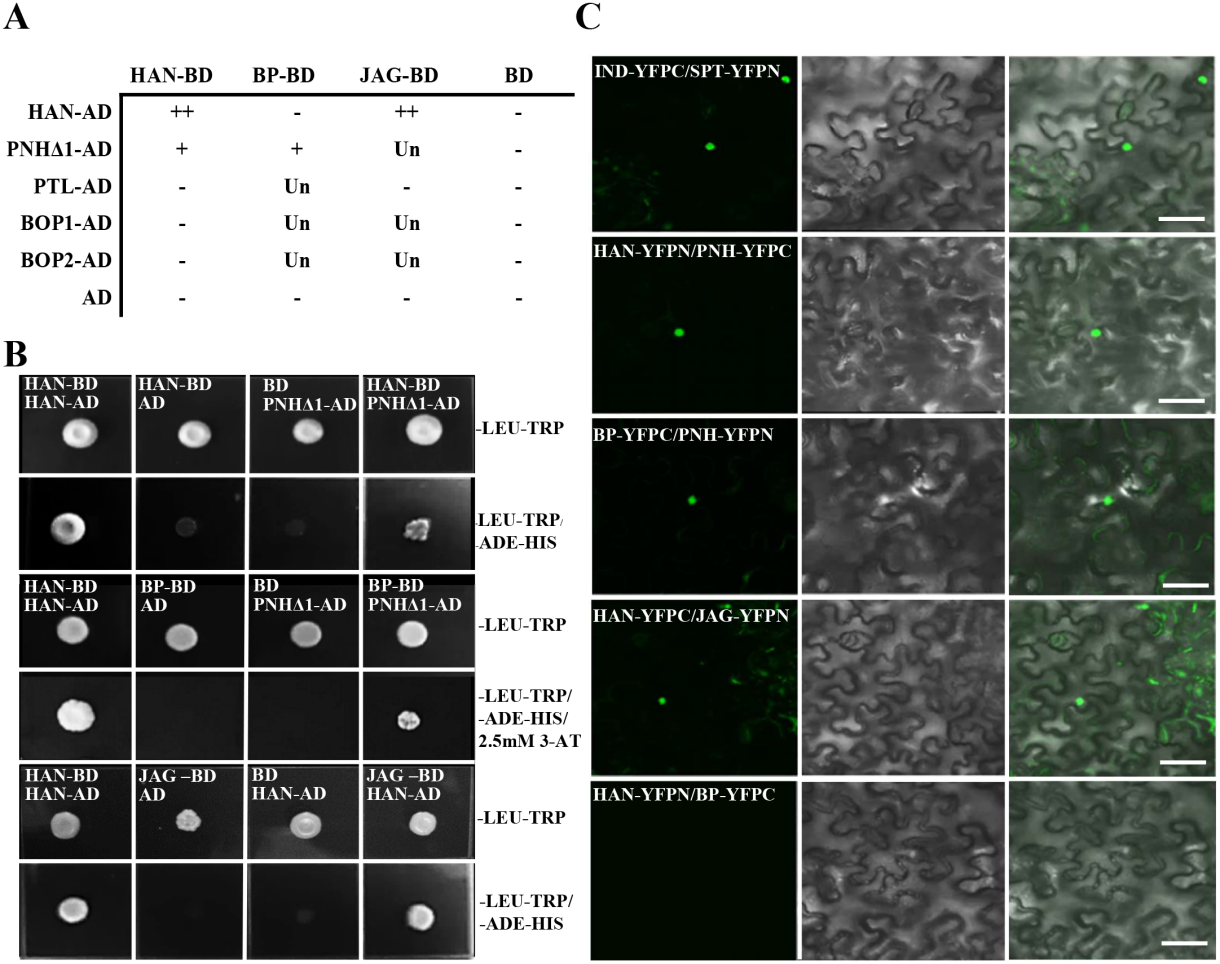
Protein interaction as detected by yeast two hybrid and bimolecular fluorescence complementation (BiFC). (A-B) Yeast two-hybrid assays. Summary of interactions performed (A).–indicates no interaction; + indicates positive interaction; ++ indicates strong interaction; Un indicates unknown. JAG-BD represents JAG fused with the GAL4 DNA binding domain (BD). HAN-AD indicates HAN fused with the GAL4 activation domain (AD). Similar labels were used for the other constructs. Mating with empty vector pGBKT7 or pGADT7 was used as a negative control. The positive control was the combination of HAN-BD and HAN-AD [49]. Yeast two-hybrid assays showed interactions between HAN and JAG, HAN and PNH, PNH and BP (B). Clones grown on medium lacking LEU-TRP indicated expression from both plasmids, and clones grown on selection medium lacking LEU-TRP and ADE-HIS suggested physical interactions between prey and bait proteins. 2.5mM 3-AT was used for inhibiting self-activation. (C) BiFC experiments show that HAN interacts with JAG and PNH, and PNH interacts with BP. Genes fused with the N-terminal or C-terminal fragment of YFP (YFPN or YFPC) were co-introduced into *Nicotiana benthamiana* leaves. INDEHISCENT (IND)-YFPC and SPATULA (SPT)-YFPN were used as a positive control [71]. A positive interaction is shown by the YFP fluorescence (green) in nuclei (left panel), differential interference contrast (DIC) of the tobacco cells is shown in the middle panel, and the two merged channels are shown in the right panel. The label IND-YFPC represents IND fused with C-terminus half of YFP in frame, and similarly for other constructs. Bars = 50μm.

Based on the genetic and transcriptional data, yeast two-hybrid assays were used to investigate possible protein interactions between HAN with the two meristem regulators (BP and PNH) and three primordia-expressed regulators (PTL, JAG, BOP1/2) (Fig 4A). Considering the possible toxicity of full length PNH to the yeast cells, a series of deletion constructs of PNH was generated to test its interaction with other proteins (Fig 4B and S5 Fig). The PNH protein can be divided into three regions: the N-terminus (part I), the PAZ domain (part II) and the MID and KIWI domains (part III) (S5A Fig) [48]. Among all the deletion constructs of PNH, the construct (PNHΔ1) without the MID and KIWI domains had the strongest interaction with HAN and BP (Fig 4B and S5B Fig). However, HAN showed no physical interactions with BP directly (S5B Fig), suggesting that HAN communicates with the meristem through effects on PNH, and PNH interacts with BP. In addition, yeast cells co-expressing full length HAN and JAG can grow on selective medium, indicating that HAN physically interacts with JAG (Fig 4B), but there is no interaction observed between HAN and PTL, HAN and BOP1/2, no interactions detected between JAG and PTL (Fig 4A and S5B Fig).

To verify the interactions between HAN, BP, PNH and JAG in planta, bimolecular fluorescence complementation (BiFC) assays were performed in the abaxial side of tobacco leaves. The results indicated interaction of HAN with PNH, HAN with JAG, and PNH with BP, confirming that HAN physically interacts with the meristem regulator PNH and primordial regulator JAG, and PNH interacts with the other meristem regulator BP in the nucleus (Fig 4C). Consistently, the BiFC assay showed no interaction between HAN and BP, or HAN and BOP1/2 in planta (Fig 4C and S6 Fig).

### HAN may maintain boundary function via modulation of hormone action

**Fig 5:**
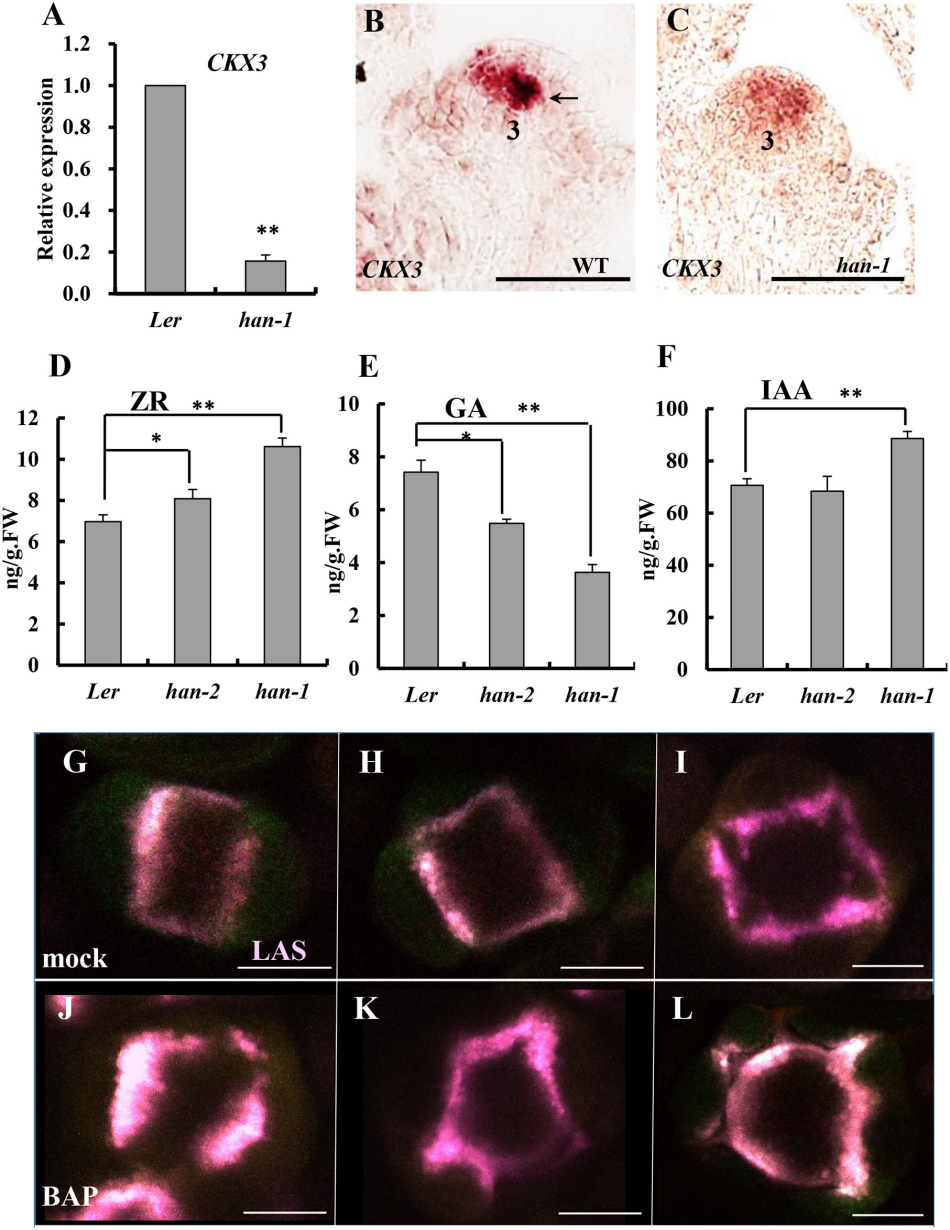
Boundary function links with phytohormone action in the inflorescence. (A) Real-time qRT-PCR analysis of RNA from the cytokinin degradation gene *CKX3* showed a substantial decrease in a *han-1* mutant background. (B-C) *in situ* hybridization indicated that *CKX3* signal is detected in the center of the floral meristem and the boundary between long stamen primordia and gynoecial primordia (black arrow) at stage 3 in a wild-type flower (B), but *CKX3* expression was reduced in level and appeared to be diffused throughout the floral meristem in the *han-1* mutant (C); (D-F) The content of *trans-*zeatin riboside (ZR) (D), gibberellins (GA) (E) and auxin (IAA) (F) in the inflorescence of *Ler*, weak allele *han-2* and null allele *han-1* plants. Three biological replicates were performed and the bars represent standard deviation. Asterisks and double asterisks represent significant difference as compared to that in the wild-type (*Ler*) at P < 0.05 and P < 0.01, respectively. Lack of asterisk indicates no significant difference (unpaired t test, P > 0.05). (G-L) Cytokinin treatment of plants harboring *pLAS*::*LAS-GFP* (purple), normal meristem-to-sepal boundary was formed in the mock-treated plants (G-I), but the boundaries display improper placement, enlarged domains as well as increased number after three days of 50μM N6-benzylaminopurine treatment (J-L). Images are representative of 20 samples. Numbers over each section represent the stage of floral development [1, 70]. Bars = 50μm.

Our previous research by time-course microarray indicated that transient induction of *HAN* by dexamethasone (DEX) treatment in the *p35S*:*HAN-GR* line led to downregulation of *HAN* through autoregulation, and specifically repressing a cytokinin degradation gene *CYTOKININ OXIDASE 3* (*CKX3*) among the CKX family [49]. To further characterize the regulation of *CKX3* by *HAN*, we examined the expression level of *CKX3* in the *han-1* null allele by qRT-PCR as well as by *in situ* hybridization (Fig 5A–5C). *CKX3* mRNA abundance was reduced more than 6-fold in the *han-1* inflorescence (Fig 5A). *In situ* hybridization showed that *CKX3* mRNA is located in the center of the FM and in the boundary between the long stamen primordia and the gynoecial primordium in WT flowers, as previously reported (Fig 5B) [50]. In the *han-1* mutant, *CKX3* signal was decreased and diffused, appearing throughout the FM (Fig 5C). Given that the expression domain of *HAN* and *CKX3* are overlapping (Figs 3C, 5B, S4E and S4G) [13], and that transient overexpression of *HAN* mimics loss of *HAN* function through self-repression [49], *HAN* may function through stimulating the expression of *CKX3* to maintain a low cytokinin level and thus reduced cell division in the boundary. Next, the content of the cytokinin *trans-*zeatin riboside (ZR) in the inflorescence was measured. As expected, the ZR levels increased in homozygotes for the *han-2* weak allele and were even higher in *han-1* null mutants as compared to WT (Fig 5D). We also measured the levels of gibberellins (GA) and auxin (IAA) in the *han* mutant inflorescence and found a significant decrease in the GA content and a small increase in the IAA level (Fig 5E and 5F).

To test whether cytokinin regulates boundary function, plants were treated with 50μM N6-benzylaminopurine (BAP) for three days, and boundary formation was observed every 24 hours by following expression of the boundary-specific reporter *pLAS*::*LAS-GFP* [51]. 50μM BAP treatment resulted in enlargement of the SAM and increased numbers of floral organs as previously reported [52]. As compared to mock-treated plants (Fig 5G–5I), meristem-to-sepal boundaries (marked by *pLAS*::*LAS-GFP* in purple) displayed improper placement, enlarged domains, and increased numbers of boundaries, which preceded and predicted the increased numbers of sepals (Fig 5J–5L). For example, as shown in Fig 5K, the formation of five boundaries predicts the development of five sepals with unequal sizes, which is often the case in cytokinin-treated lines [52].

### HAN directly binds to the *CKX3*, *JAG* and *BOP1/2*

**Fig 6:**
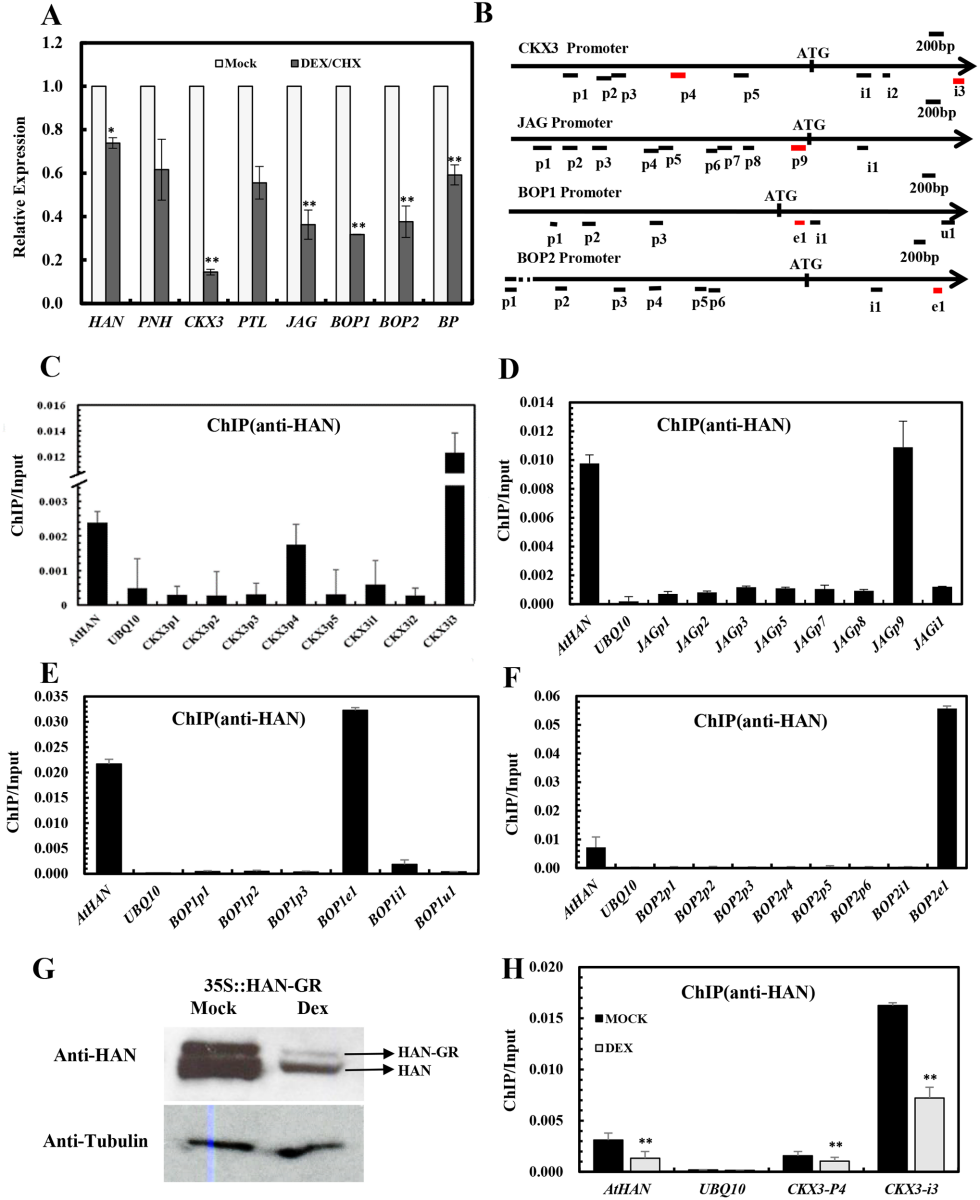
Chromatin immunoprecipitation analyses indicate that HAN directly binds to *CKX3*, *JAG* and *BOP1/2*. (A) Transcription analyses by qRT-PCR in the *p35S*:*HAN-GR* inflorescences treated with dexamethasone (DEX) and cycloheximide (CHX) for 4h. *Arabidopsis ACTIN2* was used as an internal control to normalize the expression. Three biological replicates were performed for each gene, and the bars represent the standard deviation. Asterisks and double asterisks represent significant difference as compared to that in the wild-type (*Ler*) at P < 0.05 and P < 0.01, respectively (unpaired t test). (B) Schematic diagram of the amplicons located in the *CKX3*, *JAG*, *BOP1* and *BOP2* genomic sequence used for ChIP analysis. Letter p represents promoter, i represents intron, e indicates exon, u represents UTR. (C-G) ChIP PCR assay with anti-HAN antibody showed the enrichment of amplicons from *CKX3* (C), *JAG* (D), *BOP1* (E) and *BOP2* (F) from wild-type *Ler* inflorescence. (G) Western blot analyses in the p35S::HAN-GR line showed reduced HAN protein level after induction of HAN by DEX treatment. (H) ChIP PCR assay of HAN and CKX3 in the *p35S*:*HAN-GR* inflorescences treated with DEX for 3 days. The data were the average of two biological replicates. HAN and UBQ10 were used for positive and negative controls, respectively. Double asterisks represent significant difference as compared to that in the mock-treated plants at P < 0.01 (unpaired t test).

To explore whether HAN directly regulates the transcription of *BP*, *PNH*, *PTL*, *JAG*, *BOP1/ 2* and *CKX3*, qRT-PCR was performed in the inflorescence after 4h treated with DEX and cycloheximide in 35S:HAN-GR plants. As shown in Fig 6A, the expression of *CKX3*, *JAG*, *BOP1*, *BOP2* and *BP* was significantly reduced compared to the mock-treated plants [49]. Thus, a chromatin immunoprecipitation assay (ChIP) was performed, followed by quantitative PCR analysis (ChIP-PCR), with anti-HAN antibodies, to verify the direct bindings. The specificity of anti-HAN antibodies has been previously tested [49]. The various amplicons used for the ChIP-PCR assay are shown in Fig 6B, which contained the enriched regions of DNA sequences WGATAR (W = A or T and R = A or G) in the promoters and genic regions of *CKX3*, *JAG*, *BOP1*, *BOP2* or *BP*. The promoter region −977 to −735 bp of HAN was used as a positive control and an amplicon derived from the *UBQ10* promoter was used as a negative control [49]. Amplicons CKX3p4 and CKX3i3 were significantly enriched when normalized to the negative control (Fig 6C). CKX3p4 spans the promoter region from -1677 to -1511 bp of *CKX3*, with the recognition motif WGATAR at -1619 ~ -1614bp. CKX3i3 locates in the first intron region from 1539 to 1693 bp of *CKX3*, with the recognition motif at 1569~1574 bp and the ChIP/Input ratio increased over 5-fold compared to the positive control HAN (Fig 6C). Similarly, amplicon JAGp9, which spans the promoter region from -282 to -96 bp of *JAG*, with two recognition motifs at -175~ -170 bp and -166 ~ -161 bp, respectively, was significantly enriched (Fig 6D). BOP1e1 and BOP2e1, which span the first exon from 330 to 476 bp of *BOP1* (recognition motif at 363~368 bp), and the second exon region from 1862 to 2012 bp of *BOP2* (recognition motif at 1939~1944bp), respectively, were also significantly enriched, with the ChIP/Input ratio increased 1.5 and 7.7 fold, respectively, as compared to the positive control HAN (Fig 6E and 6F). By contrast, all of the other tested amplicons from *CKX3*, *JAG* or *BOP1/2* were not enriched compared to the *UBQ10* amplicon, suggesting that the ChIP-PCR assay was amplicon-specific. Further, no amplicons in the promoters and genic regions of *BP*, *PNH* and *PTL* were found to be significantly enriched (S7 Fig), indicating that HAN did not directly bind to *BP*, *PNH* and *PTL*.

Given that the expression of *CKX3*, *JAG* and *BOP2* were greatly reduced in both *han-1* and DEX-treated 35S:HAN-GR plants, we verified the *HAN* autoregulation by western blotting and the binding of HAN and CKX3 using ChIP-PCR between the DEX- and mock-treated 35S:HAN-GR plants. Our data showed that HAN protein was greatly reduced in the 35S::HAN-GR line upon DEX treatment, supporting the self-regulation of HAN (Fig 6G). Consistently, binding on CKX3 and on HAN itself was significantly reduced upon DEX treatment (Fig 6H).

## Discussion

### HAN communicates with cells in the meristem through PNH, and with organ primordia via JAG and BOP2 to precisely orchestrate flower development

**Fig 7:**
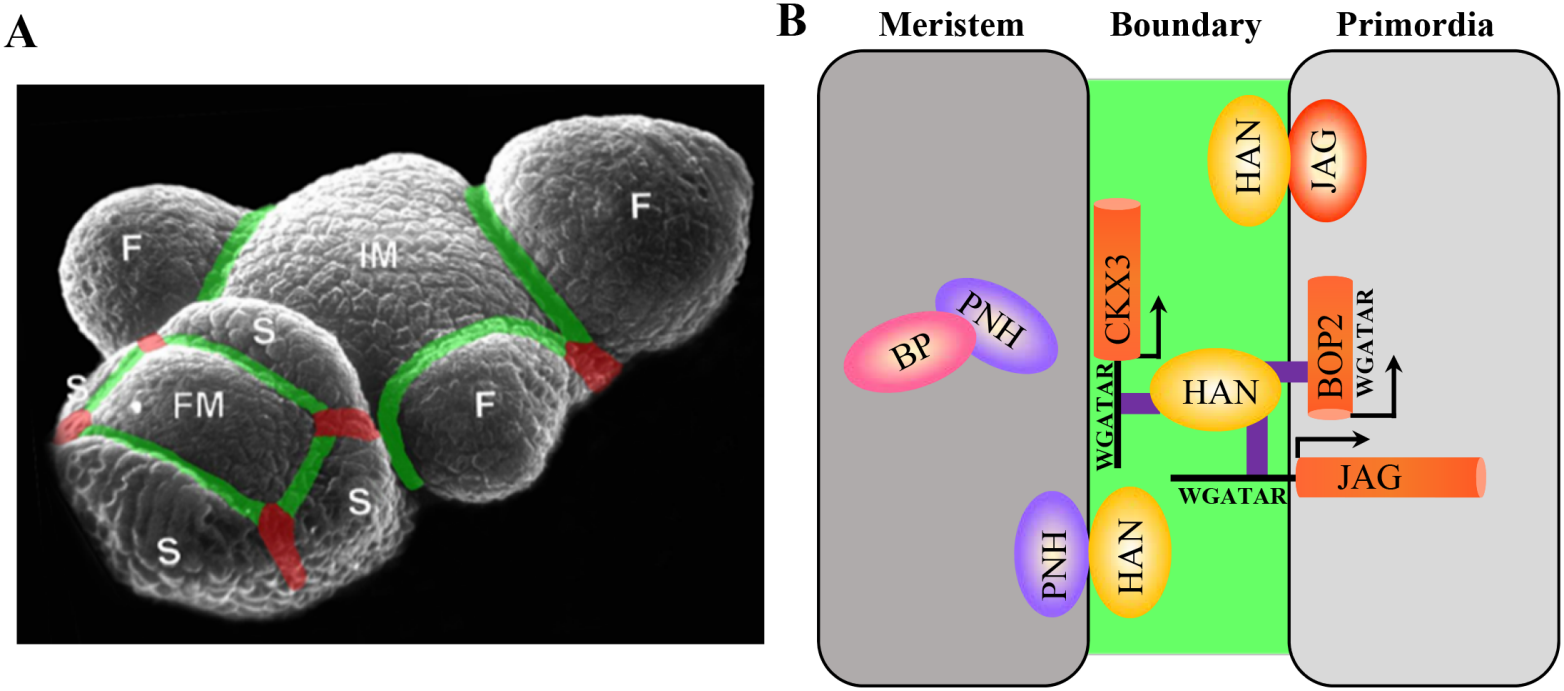
Regulatory interactions between boundary, meristem and floral organ primordia in *Arabidopsis*. (A) Schematic boundaries in the *Arabidopsis* inflorescence. The M–O boundaries were marked in green, and the O–O boundaries were marked in red. IM, inflorescence meristem; F, floral primordia; FM, floral meristem; S, sepal primordia. (B) Working model of boundary gene *HAN* serving as a link between the meristem and floral primordia to separate distinct cell identity during flower development. The boundary communicates with meristem through a HAN-PNH interaction, and PNH interacts with BP in the meristem to maintain meristem organization during continuous organogenesis. On the other hand, HAN physically interacts with JAG protein and directly binds to the promoter of *JAG* and genic region of *BOP2* to promote floral organ development. In addition, HAN directly activates *CKX3* to reduce cytokinin content, thus suppressing cell division in the boundary and serving as a bridge between meristem and organ primordia.

Proper boundary formation is required for meristem maintenance, organ separation, floral organ patterning, and axial meristem initiation [10, 11, 14, 16, 19, 20, 22, 53]. Previous studies have shown that boundary-expressing *CUC* genes induce the expression of the meristematic marker *STM*, while *STM* represses *CUC* expression in the meristem, forming a negative feedback loop during embryogenesis [9, 22]. Here we found that the boundary-expressing gene *HAN* interacts with meristem regulators *PNH* genetically, transcriptionally and biochemically. Double mutant *han-2 pnh-2* displayed synergistic effect on petal reduction and meristem organization (Figs 1 and 2). At the transcriptional level, *HAN* and *PNH* promote each other, while *HAN* represses *BP*, and *BP* represses *PNH* (Fig 3). At the protein level, HAN interacts with PNH and PNH interacts with BP (Fig 4). Therefore, HAN may communicate with the meristem through a direct interaction with PNH and indirectly with BP to ensure proper meristem organization and flower development (Fig 7B). The expression of *HAN* and *PNH* overlap in the boundary regions and the bottom of the meristem in the stage 2 flowers (Fig 3C and 3G) [13], the interaction between HAN and PNH may occur in these overlapping regions to maintain proper meristem organization during continuous organogenesis (Fig 7B).

On the other side, the boundary gene *HAN* communicates with floral organ primordia through *JAG* and *BOP2*. Genetically, *HAN* coordinatively regulates flower organ development with *JAG* and *BOP1/2* (Fig 1 and S1 Fig). qRT-PCR analysis and *in situ* hybridization showed that *HAN* promotes the expression of *JAG* and *BOP2* in the organ primordia (Fig 3). Biochemical analysis showed that HAN physically interacts with JAG (Fig 4), and a ChIP-PCR assay indicated that HAN directly binds to the promoter of *JAG* and exon of *BOP2* (Fig 6). Therefore, *HAN* directly stimulate the transcription of *JAG* and *BOP2*, and interact with JAG at the protein level (Fig 7B). Given that transcripts of *HAN*, *JAG* and *BOP2* overlap in the boundary regions in the stage 2 flower (Fig 3C, 3M and 3Q), HAN may directly stimulate the transcription of *JAG* and *BOP2* in the boundary region to promote organ primordia development. Consistent with this notion, the serrated sepals in the *jag* mutant were also observed in a *han-1* mutant, which could be due to reduced *JAG* expression, or to elimination of its protein partner, in the *han-1* mutant [38]. Previous finding showed that JAG can directly bind to the promoter of *BOP2* [54]. Our data showed that *BOP2* was significantly reduced in the *jag-3* mutant, indicating that JAG directly stimulates the transcription of *BOP2* in the inflorescence. Given that HAN directly binds to the exon of *BOP2*, HAN promotes the expression of *BOP2* directly or indirectly through *JAG*. Despite HAN also directly binds to the exon of *BOP1* (Fig 6E), transcription of *BOP1* was not changed in the *han-1* mutant, suggesting that additional factors may antagonize the effect of HAN on *BOP1*. In the *bop1 bop2* mutant, *JAG* expression in the inflorescence is down-regulated, in contrast to the upregulation of *JAG* expression in leaves or in the vegetative shoot apex [40], indicating that different interaction modules of *JAG* and *BOP1/2* exist during leaf and flower development.

Previous data showed that JAG directly binds to the promoter of *PTL* to control petal growth and shape [55], and we found that *JAG* repressed the expression of *PTL* (S3C Fig). Thus, the role of HAN in control of petal number and petal morphology as revealed by the double mutant analysis (Fig 1) can be explained by the direct interaction with JAG and BOP2 in the petal boundaries, and thus indirect interaction with PTL during flower development in *Arabidopsis*. Notably, ChIP-Seq showed that JAG directly targets *HAN* as well [54]. However, the expression of *HAN* showed no significant change in the *jag-3* mutant (Fig 3A), probably due to autoregulation of *HAN* [49].

### CKX3 and GNC/GNL may act as the direct linkage between HAN and hormone actions

Our qRT-PCR, *in situ* hybridization and ChIP-PCR data showed that HAN directly binds to the cytokinin degradation gene *CKX3* and promotes *CKX3* expression (Figs 5 and 6). In the *han-1* mutant, the *CKX3* signal intensity was reduced and diffused throughout the FM (Fig 5C), resulting in elevated cytokinin content (Fig 5D). Exogenous cytokinin treatment disrupts boundary formation and results in increased floral organ numbers (Fig 5G–5L). Therefore, *HAN* may maintain the proper boundary function by directly activating *CKX3*, thus reducing cytokinin content and suppressing cell division in the boundary (Fig 7B). Han et al. [56] showed that pAP1::IPT8 (which encodes a rate-limiting enzyme in cytokinin biosynthesis) lines displayed loss of petals [56], while loss of function of both *CKX3* and *CKX5* results in a slight increase in the number of sepals and petals[50], rather than the reduced floral organ number observed in the *han-1* mutant, suggesting that the distribution of cytokinin rather than the content of cytokinin in the flower is more essential for regulation of petal number, and that HAN regulates flower development via a complex interaction network, with the CKX3-mediated cytokinin pathway only as one branch.

In addition, the signal intensity of the auxin response marker DR5 was previously shown to be greatly reduced in the *han-2* mutant [49], while the IAA level was up-regulated in the *han* mutant (Fig 5F), suggesting that *HAN* represses auxin biosynthesis and promotes auxin signaling in the inflorescence. Consistent with the antagonistic interaction between auxin and GA [57], the GA level was significantly decreased in the *han* mutant inflorescence (Fig 5E). Recently, HAN was shown to repress itself and three GATA3 family genes, *HAN-LIKE 2* (*HANL2*), *GATA*, *NITRATE-INDUCIBLE*, *CARBON-METABOLISM-INVOLVED* (*GNC*), and *GNC-LIKE* (*GNL*) [49]. *GNC* and *GNL* are direct downstream targets of *AUXIN RRESPONSE FACTOR2* (*ARF2*) that mediates auxin response, and *GNC* and *GNL* are also downstream targets of the GA signaling pathway involving DELLAs and PIFs in *Arabidopsis* [58]. Therefore, HAN may regulate flower development through the CKX3-mediated cytokinin homeostasis, auxin and GA biosynthesis, and GNC/GNL-mediated auxin and gibberellin responses.

## Methods

### Plant materials and genetics

The *Arabidopsis thaliana* Landsberg erecta (*Ler*) and Columbia (*Col*) ecotypes, the mutant alleles *han-1* (*Ler)*, *han-2* (*Ler)*, *pnh-2 (Ler)*, *jag-3 (Ler)*, *bp-1 (Ler)*, *han-2(Col)* and *ptl-1(Col)* were described previously [11, 13, 26, 38, 59] and obtained from the Meyerowitz lab stock collection. The reporter line *pLAS*::*LAS-GFP* was kindly provided by Dr. Yuval Eshed [51]. The *bop1-4 bop2-11* double mutant plants (*Col*) were kindly provided by Jennifer C. Fletcher. Double or triple mutant combinations with *han-2* were generated by crossing using the same ecotype background, and identified by genotyping using the primers listed in S1 Table. For *han-2* genotyping, a 852-bp fragment was amplified and digested by TseI, which recognizes the mutant site. For *jag-3* genotyping, PCR products from the mutant were cleaved by TseI. For *pnh-2* genotyping, a 111-bp product was amplified by PCR, and EcoRIcleaves only the wild-type product. For *ptl-1* genotyping, a 726-bp fragment was amplified and digested by CfrI, which digests only the wild-type product. Genotypings for *bop1-4 bop2-11* and *bp-1* were performed as described previously [44]. Plants were grown in soil at 22°C under conditions of 16h light/8h dark.

### Quantitative real-time PCR

Total RNA was isolated from 3–5 inflorescence samples using RNaEXTM Total RNA Isolation Solution (Generay, China). cDNA was synthesized from 4μg total RNA using reverse transcriptase (Aidlab, China) and qRT-PCR analyses were performed on an ABI PRISM 7500 Real-Time PCR System (Applied Biosystems, USA). Each qRT-PCR experiment was performed in three biological replicates and three technical replicates. The *ACTIN2* gene was used as an internal reference to normalize the expression data. Fold change was calculated using the 2^-ΔΔCt^ method [60] and the standard deviation was calculated between three biological replicates, using the average of the three technical replicates for each biological sample. The gene-specific primers are listed in S1 Table.

### Endogenous hormone measurement

To examine auxin, cytokinin and gibberellin levels in the *han-1* and *han-2* mutant plants, about 0.1g of inflorescence (about 20–35 inflorescence) was harvested from *han-1*, *han-2* or *Ler* plants grown under the same conditions and immediately frozen in liquid nitrogen until further use. Sample extraction and hormone measurements were performed using enzyme-linked immunosorbent assays as previously reported [61]. Standard IAA, GA and trans-zeatin riboside (ZR) (Sigma, USA) was used for calibration.

### *in situ* hybridization

*Arabidopsis* inflorescences were fixed in 3.7% formol-acetic-alcohol (FAA) (3.7% formaldehyde, 5% glacial acetic acid, and 50% ethanol) and stored at 4°C until use. Probe synthesis was performed on cDNA using gene-specific primers including SP6 and T7 RNA polymerase binding sites. Probes for *HAN*, *PNH*, *BOP1/2*, *PTL*, and *CKX3* were made using the same sequences as previously reported [13, 40, 44, 50, 59, 62], and probes for *JAG* and *BP* were synthesized with the specific coding sequence fragments as templates. Sample fixation, sectioning and *in situ* hybridization was performed as previously described [49]. The primers for probe synthesis are listed in S1 Table.

### DEX and cytokinin treatments

Transient overexpression of *HAN* was achieved through 10μM DEX treatment on *p35S*::*HAN-GR* inflorescence apices. 10μM cycloheximide was used with 10μM DEX for 4h treatment. DEX solution was applied by pipette every 24h. Cytokinin treatment was performed using 50μm N6-benzylaminopurine, and applied by pipette every 24h. Each treatment was repeated at least three times with corresponding mock-treated controls.

### Live imaging

Plants were grown and inflorescence meristems were prepared for live imaging as previously described [6]. All imaging was done using a Zeiss 510 Meta laser scanning confocal microscope with a 40x water dipping objective using the Z-stacks mode. For the *pLAS*::*LAS-GFP* reporter line, 20 samples were imaged to confirm the observed patterns were representative, and similar sets of lasers and filters were used to image the reporter as previously described [6, 63].

### Yeast two hybrid assay

Full-length coding sequences for *HAN*, *JAG*, *PNH*, *BP*, *IND*, *SPT*, *PTL*, *BOP1*, *BOP2* or a series of truncated *PNH* fragments were cloned into pGBKT7 (bait vector) or pGADT7 (prey vector). All constructs were confirmed by sequencing before transformation into yeast strain AH109. The bait and prey vectors were transformed according to the manufacturer’s instructions of Matchmaker^TM^ GAL4 Two-Hybrid System 3 & Libraries (Clontech). Protein interactions were assayed on selective medium lacking Leu, Trp, His and Ade or supplemented with 2.5 mM 3-Amino-1, 2, 4-triazole (3-AT). The gene primers used for yeast two hybrid experiments are listed in S1 Table.

### Bimolecular Fluorescence Complementation (BiFC) assay

Full-length coding sequences for *HAN*, *JAG*, *PNH*, *BP*, *IND*, *SPT*, *BOP1* and *BOP2* (without stop codons) were amplified by PCR using gene-specific primers, and cloned into the vectors pSPYNE-35S or pSPYCE-35S containing each half of YFP (N- or C- terminus) to generate the fusion proteins (such as *HAN*-YFP N-terminus) in frame as previously described [64]. All constructs were verified by sequencing before transformation into *Agrobacterium tumefaciens* strain GV3101. The two plasmids for testing specific interaction were co-transformed into the abaxial sides of 4-7-week old *Nicotiana benthamiana* leaves as previously described [65]. After 48h co-infiltration, the tobacco leaves were imaged using a Zeiss LSM 510 Meta confocal laser scanning microscope. YFP signals and DIC of tobacco cells were taken at the same time from different detection channels. The gene primers used for BiFC are listed in S1 Table.

### Chromatin immunoprecipitation

ChIP-PCR was performed as described by Gendrel et al. [66] with slight modifications. Briefly, about 2g of inflorescence tissue from wild-type *Ler* or DEX-treated *p35S*::*HAN-GR* line for three days were harvested and fixed in 37ml 1% formaldehyde and cross-linked for 15 min with vacuum infiltration at room temperature, followed by addition of glycine with vacuum infiltration for 5 min to terminate the cross-linking reaction. Nuclei were isolated and lysed, and chromatin was sonicated to an average size of 500 bp. The sonicated chromatin served as input and stored at -20°C until use. Immunoprecipitation reactions were performed using anti-HAN antibody [49] and no antibody as a negative control. The complex of chromatin-antibody was captured with protein G agarose beads (Millipore) followed by precipitated DNA purification and elution, and DNA deposited with glycogen carrier (Thermo) served as a template for qRT-PCR. The enrichment regions of DNA sequences WGATAR (W = A or T and R = A or G) in the promoters or genic regions were chosen to perform qRT-PCR [67–69]. Two biological repeats and three technical replicates were performed for each gene. HAN and UBQ10 were used for positive and negative controls, respectively [49]. The ChIP/Input ratio was calculated by the equation 2^(Ct(MOCK)-Ct(HAN-ChIP))^/2^(Ct(MOCK)-Ct(INPUT))^. The primer pairs used in ChIP-PCR were listed in S1 Table.

### Scanning electron microscopy

Inflorescence of *Ler*, *han-2*, *bp-1*, *han-2 bp-1*, *pnh-2*, *han-2 pnh-2* from 40-day-old plants, and stage 7–9 fruit samples of *han-2*, *bop1bop2* and *han-2bop1bop2* were prepared for SEM. After removing the flowers or floral organs, samples were fixed in FAA overnight. The samples were then critical-point dried in liquid CO_2_, sputter coated with gold and palladium for 60s, and examined at an acceleration voltage of 2kV using a scanning electron microscope (Hitachi Model S-4700, Japan).

### Western blotting assay

Inflorescence tissues from mock or DEX-treated *p35S*::*HAN-GR* line for three days were harvested in liquid nitrogen. The plant total protein extraction kit (Sigma-Aldrich) was used for protein extraction. Western blotting was performed as previously described [49].

### Accession numbers

Sequence data from this article can be found in the *Arabidopsis* Genome Initiative or GenBank/EMBL databases under the following accession numbers: *HAN* (AT3G50870), *PNH* (AT5G43810), *BOP1* (AT3G57130), *BOP2* (AT2G41370), *JAG* (AT1G68480), *PTL* (AT5G03680), *KNAT1/BP* (AT4G08150), *CKX3* (AT5G56970), *IND* (AT4G00120), *SPT* (AT4G36930).

## Supporting Information

S1 Fig Inflorescences phenotypes of mutations in *HAN* and genes regulating meristem and floral organ development. (A-C) Lateral view of inflorescences in *han-2* (A), *bp-1* (B) and *han-2 bp-1* double mutant (C); (D-O) Top view of the representative inflorescences of *Ler* (D), *han-2 (Ler)* (E), *pnh-2* (F), *han-2 pnh-2* (G), *Col* (H), *han-2(Col)* (I), *ptl-1* (J), *han-2 ptl-1* (K), *jag-3* (L), *han-2 jag-3* (M), *bop1bop2* (N), *han-2 bop1 bop2* (O). Bars = 1mm.
(TIF)

S2 Fig Abscission and rosette leaves phenotypes of mutations in *HAN* and *BOP* genes. *(A) han-2 bop1 bop2* triple mutants retain floral organs in the siliques as in a *bop1 bop2* double mutant. Bars = 1cm. (B) Scanning electron micrographs of the petal abscission zones (AZs). *han2 bop1 bop2* flowers lack AZs as in *bop1 bop2*. Bars = 100μm. (C) Morphology of each rosette leaf of *Col*, *han-2*, *han-2 bop1 bop2* and *bop1 bop2* leaves. Bars = 1cm.
(TIF)

S3 Fig Relative transcript levels of *BP*, *PNH*, *PTL*, *JAG*, *BOP1*, and *BOP2* in wild-type and in the respective mutant backgrounds. (A-F) Transcription analyses by qRT-PCR of *BP* (A), *PNH* (B), *PTL* (C), *JAG* (D), *BOP1* (E) and *BOP2* (F) in inflorescences of different mutants.
(TIF)

S4 Fig RNA localization of *HAN*, *BP*, *PTL* and *BOP1/2* as detected by *in situ* hybridization. (A-H) Expression of *HAN* in wild-type (A, E, G), *pnh-2* (B), *bop1 bop2* (C), *bp-1* (D), *ptl-1*(F) and *jag-3* (H) mutant flowers. (I-J) Expression of *BP* in wild-type (I) and *han-2* mutant (J). (K-L) *PTL* is expressed in the base of sepal primordia in wild-type (K) and *han-1* (L). (M-P) *BOP1/2* signal is detected in the base of sepal primordia in wild-type (M, O) and *han-2* (N, P). Numbers over each section represent the stage of floral development [1, 70]. Bars = 50μm.
(TIF)

S5 Fig Yeast two-hybrid assays that showed weak or no interactions. (A) Schematic view of the PNH fragments used in yeast two hybrid experiments. PNH contains a variable N-terminal domain (I), a PAZ domain (II), a MID and PIWI domain (III). Δ1 (Iand II) and Δ2 (IIand III) are indicated. (B) The label HAN-BD stands for the HAN fused with the GAL4 DNA binding domain (BD), and similarly for the other constructs. Clones grown on medium lacking LEU-TRP indicated expressing both plasmids, and clones grown on selection medium lacking LEU-TRP and ADE-HIS suggested physical interactions between prey and bait proteins.
(TIF)

S6 Fig BiFC assays that showed no interactions and the negative controls. The label SPT-YFPN represents the SPT fused with N-terminus half of YFP in-frame, and similarly for other constructs. A positive interaction was shown by YFP fluorescence (green) in nuclei (left panel). Differential interference contrast images of the tobacco cells are shown in the middle panel, and the two channels merged are shown in the right panel. Bars = 50μm.
(TIF)

S7 Fig Chromatin immunoprecipitation analyses indicate no binding between *HAN* and *BP*, *PNH* or *PTL*. (A) Schematic diagram of the amplicons located in the *BP*, *PNH* and *PTL* genomic sequence used for ChIP analyses. Letter p represents promoter, i represents intron, e indicates exon, u represents UTR. (B-D) ChIP PCR assay with anti-HAN antibody showed no enrichment of amplicons from *BP* (B), *PNH* (C) and *PTL* (D in wild-type *Ler* inflorescence. The data were the average of two biological replicates. HAN and UBQ10 were used for positive and negative controls, respectively.
(TIF)

S1 Table Primer information used in this study. (DOCX)
