# Supplementary figures and images for: HANABA TARANU (HAN) Bridges Meristem and Organ Primordia Boundaries through PINHEAD, JAGGED, BLADE-ON-PETIOLE2 and CYTOKININ OXIDASE 3 during Flower Development in Arabidopsis
Source: PLoS Genet. 2015 Sep 21;11(9):e1005479. doi: 10.1371/journal.pgen.1005479 (PMC4577084; doi:10.1371/journal.pgen.1005479)

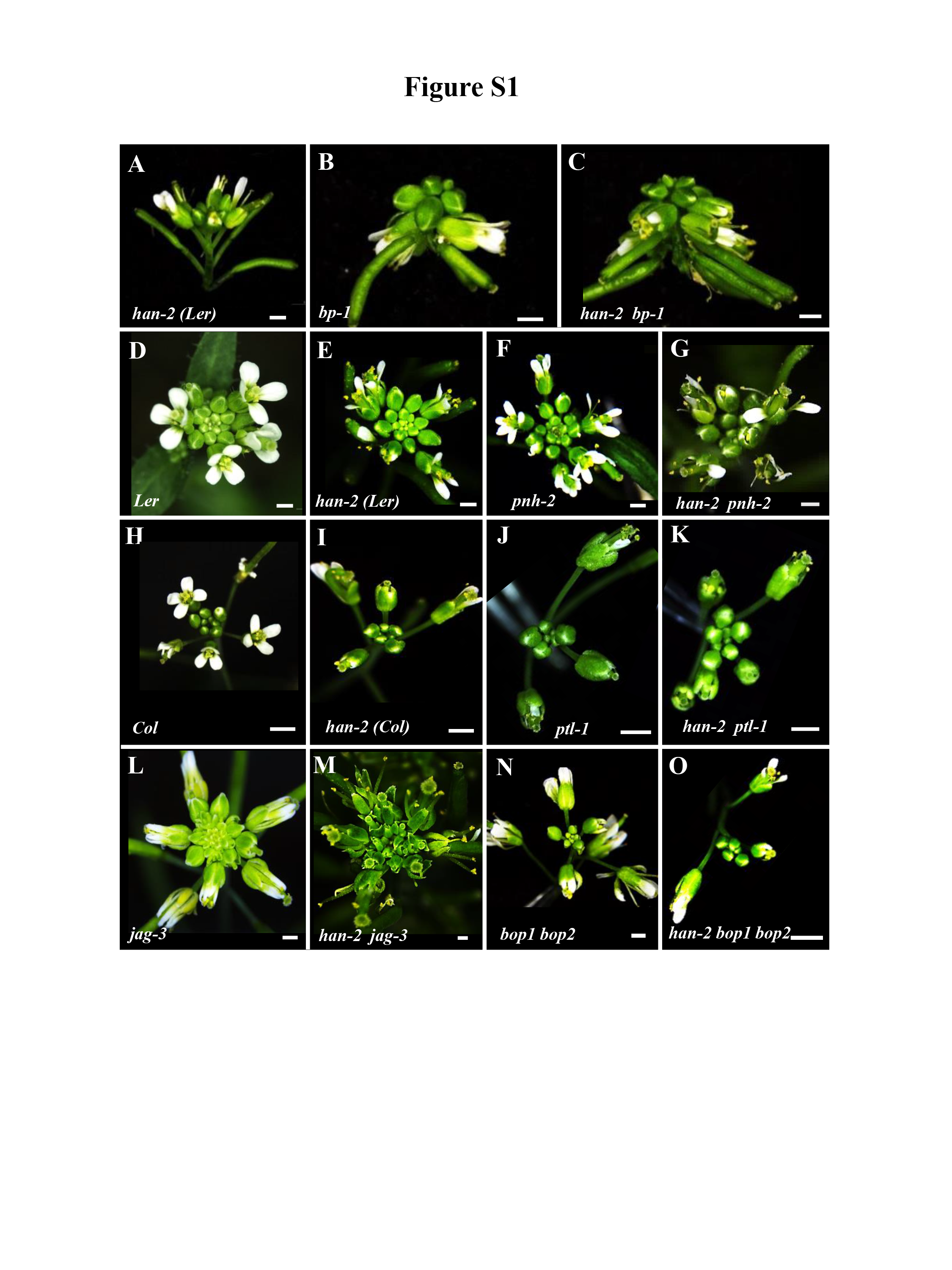

Supplement: S1 Fig — (A-C) Lateral view of inflorescences in han-2 (A), bp-1 (B) and han-2 bp-1 double mutant (C); (D-O) Top view of the representative inflorescences of Ler (D), han-2 (Ler) (E), pnh-2 (F), han-2 pnh-2 (G), Col (H), han-2(Col) (I), ptl-1 (J), han-2 ptl-1 (K), jag-3 (L), han-2 jag-3 (M), bop1bop2 (N), han-2 bop1 bop2 (O). Bars = 1mm. (TIF) [file pgen.1005479.s001.tif]

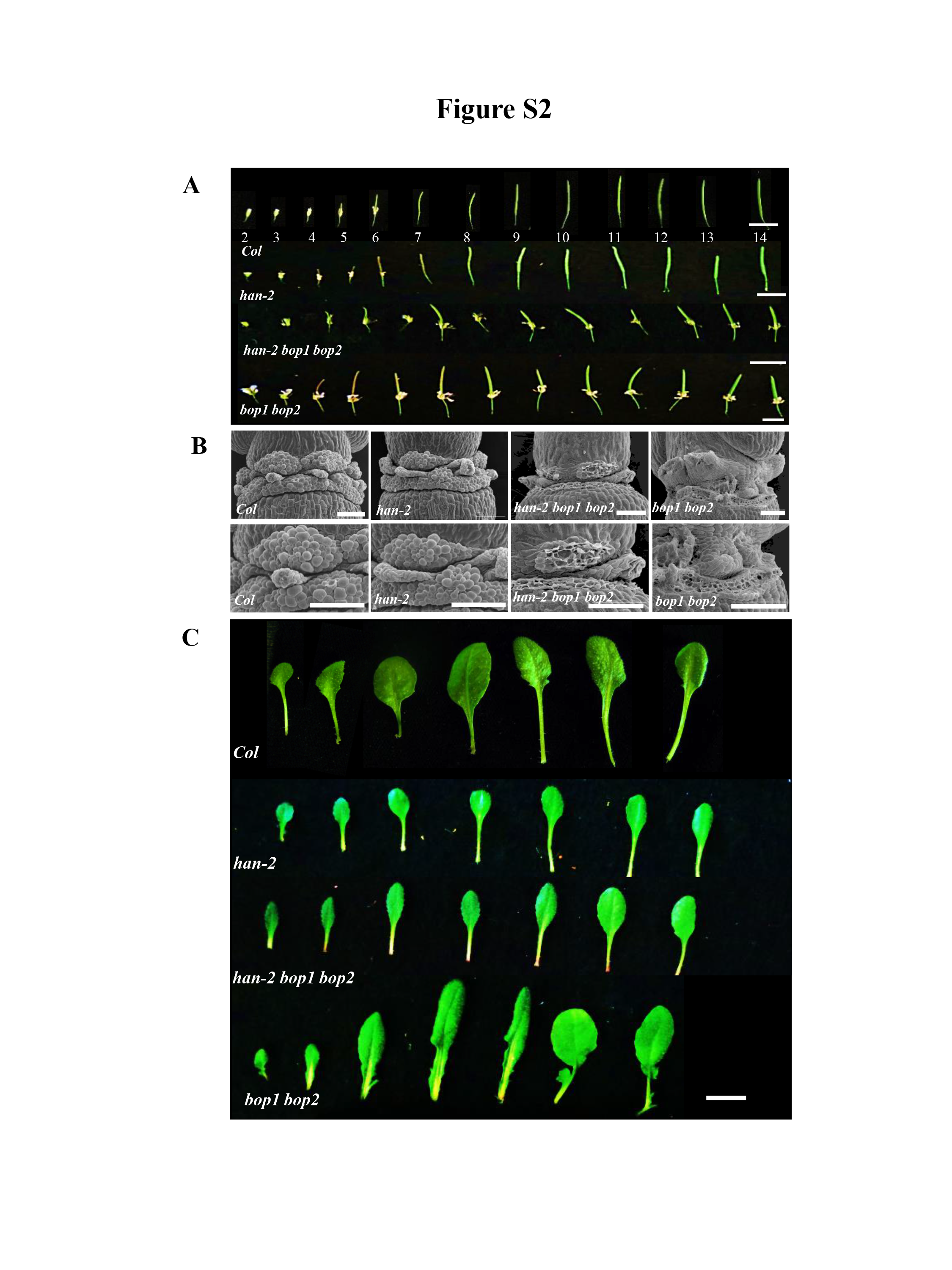

Supplement: S2 Fig — (A) han-2 bop1 bop2 triple mutants retain floral organs in the siliques as in a bop1 bop2 double mutant. Bars = 1cm. (B) Scanning electron micrographs of the petal abscission zones (AZs). han2 bop1 bop2 flowers lack AZs as in bop1 bop2. Bars = 100μm. (C) Morphology of each rosette leaf of Col, han-2, han-2 bop1 bop2 and bop1 bop2 leaves. Bars = 1cm. (TIF) [file pgen.1005479.s002.tif]

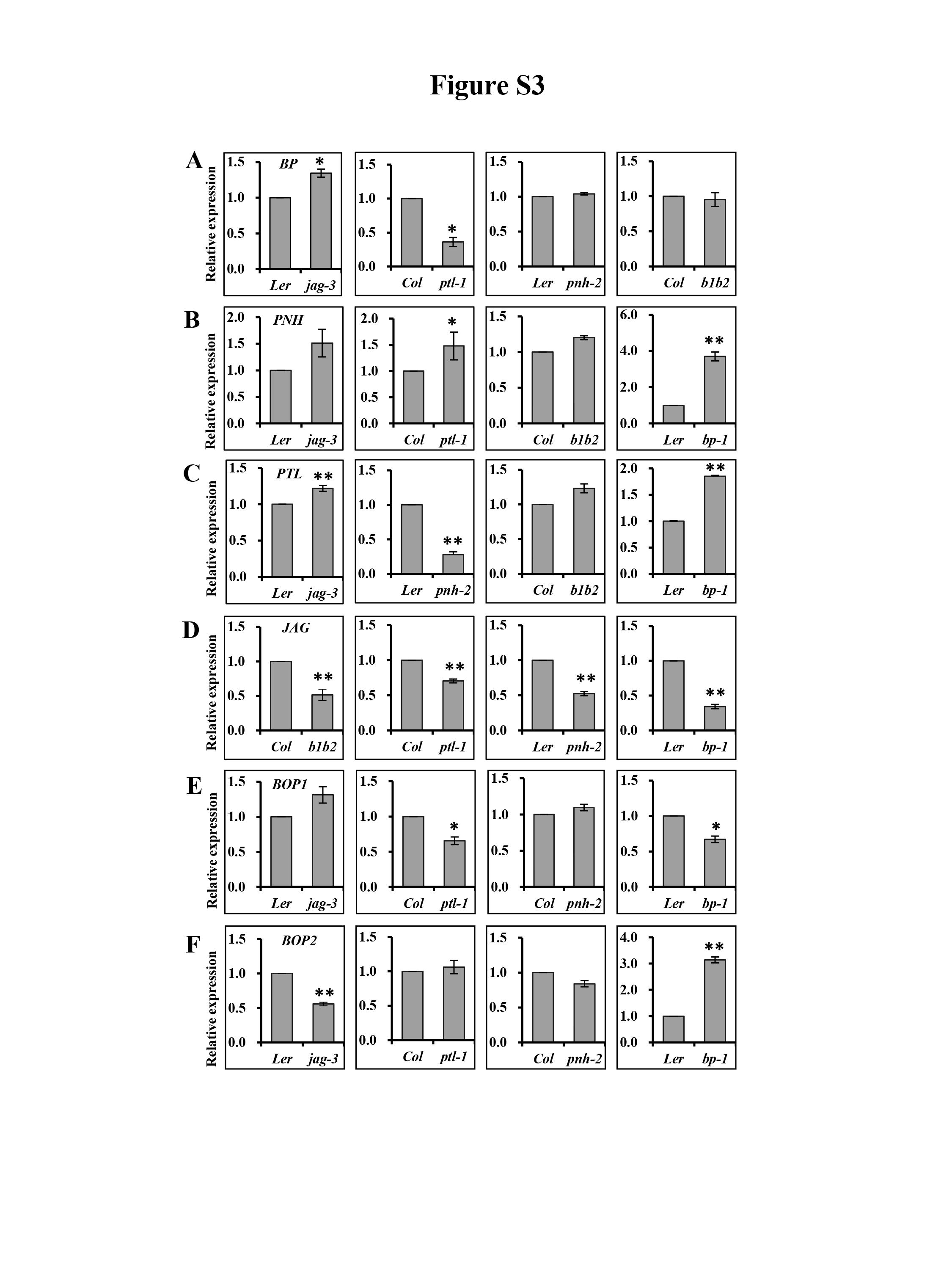

Supplement: S3 Fig — (A-F) Transcription analyses by qRT-PCR of BP (A), PNH (B), PTL (C), JAG (D), BOP1 (E) and BOP2 (F) in inflorescences of different mutants. (TIF) [file pgen.1005479.s003.tif]

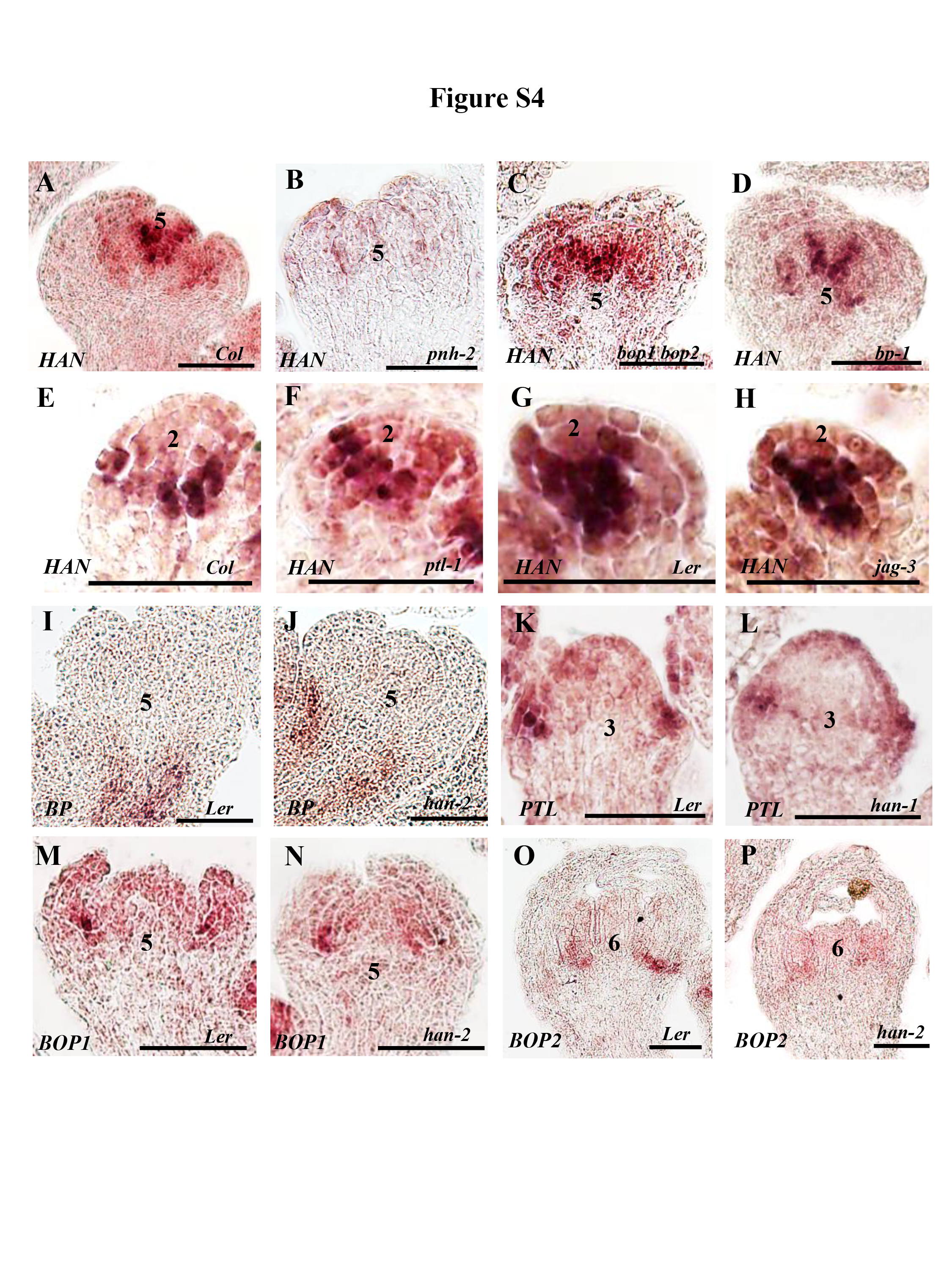

Supplement: S4 Fig — (A-H) Expression of HAN in wild-type (A, E, G), pnh-2 (B), bop1 bop2 (C), bp-1 (D), ptl-1(F) and jag-3 (H) mutant flowers. (I-J) Expression of BP in wild-type (I) and han-2 mutant (J). (K-L) PTL is expressed in the base of sepal primordia in wild-type (K) and han-1 (L). (M-P) BOP1/2 signal is detected in the base of sepal primordia in wild-type (M, O) and han-2 (N, P). Numbers over each section represent the stage of floral development [1, 70]. Bars = 50μm. (TIF) [file pgen.1005479.s004.tif]

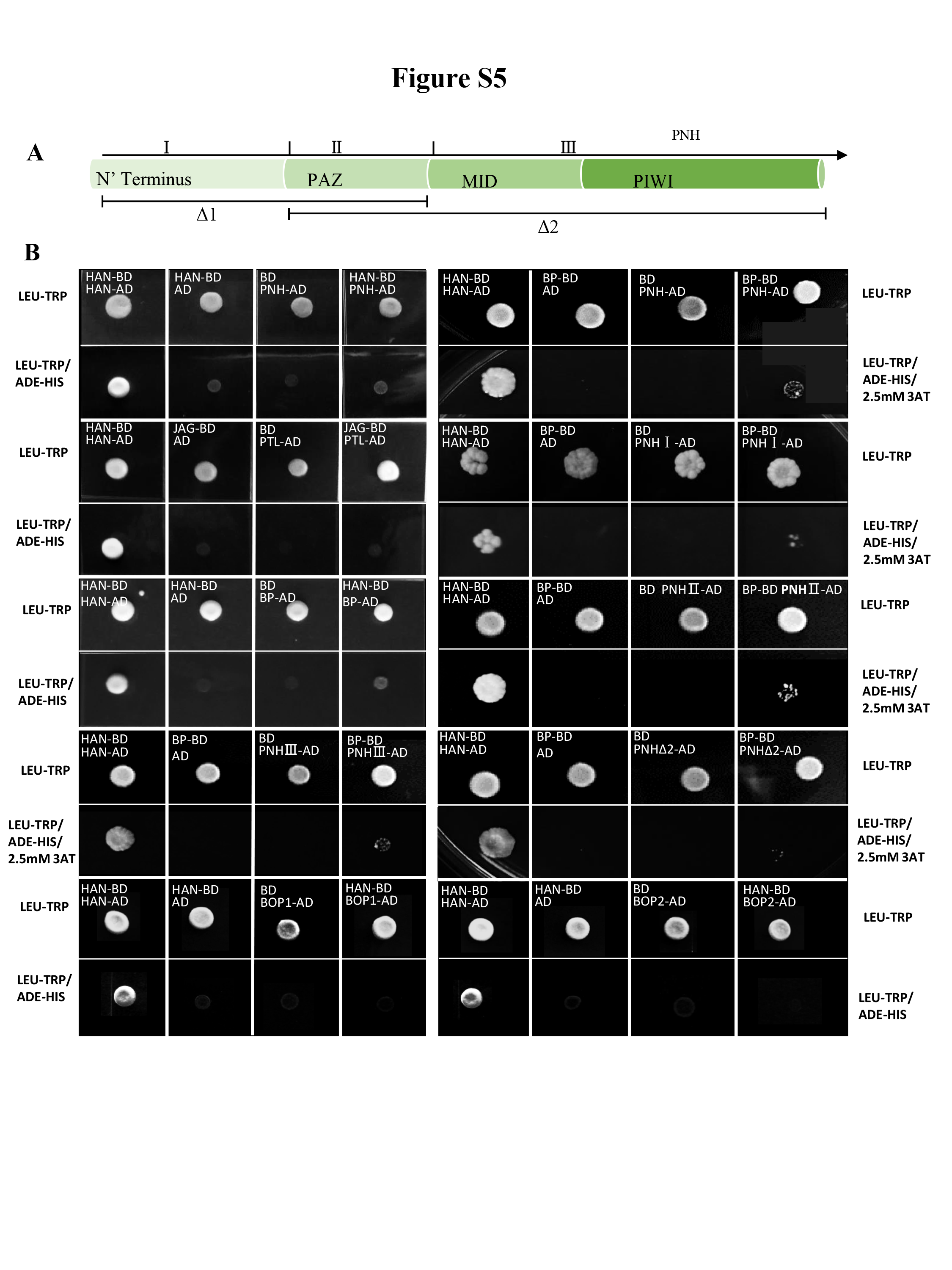

Supplement: S5 Fig — (A) Schematic view of the PNH fragments used in yeast two hybrid experiments. PNH contains a variable N-terminal domain (I), a PAZ domain (II), a MID and PIWI domain (III). Δ1 (Iand II) and Δ2 (IIand III) are indicated. (B) The label HAN-BD stands for the HAN fused with the GAL4 DNA binding domain (BD), and similarly for the other constructs. Clones grown on medium lacking LEU-TRP indicated expressing both plasmids, and clones grown on selection medium lacking LEU-TRP and ADE-HIS suggested physical interactions between prey and bait proteins. (TIF) [file pgen.1005479.s005.tif]

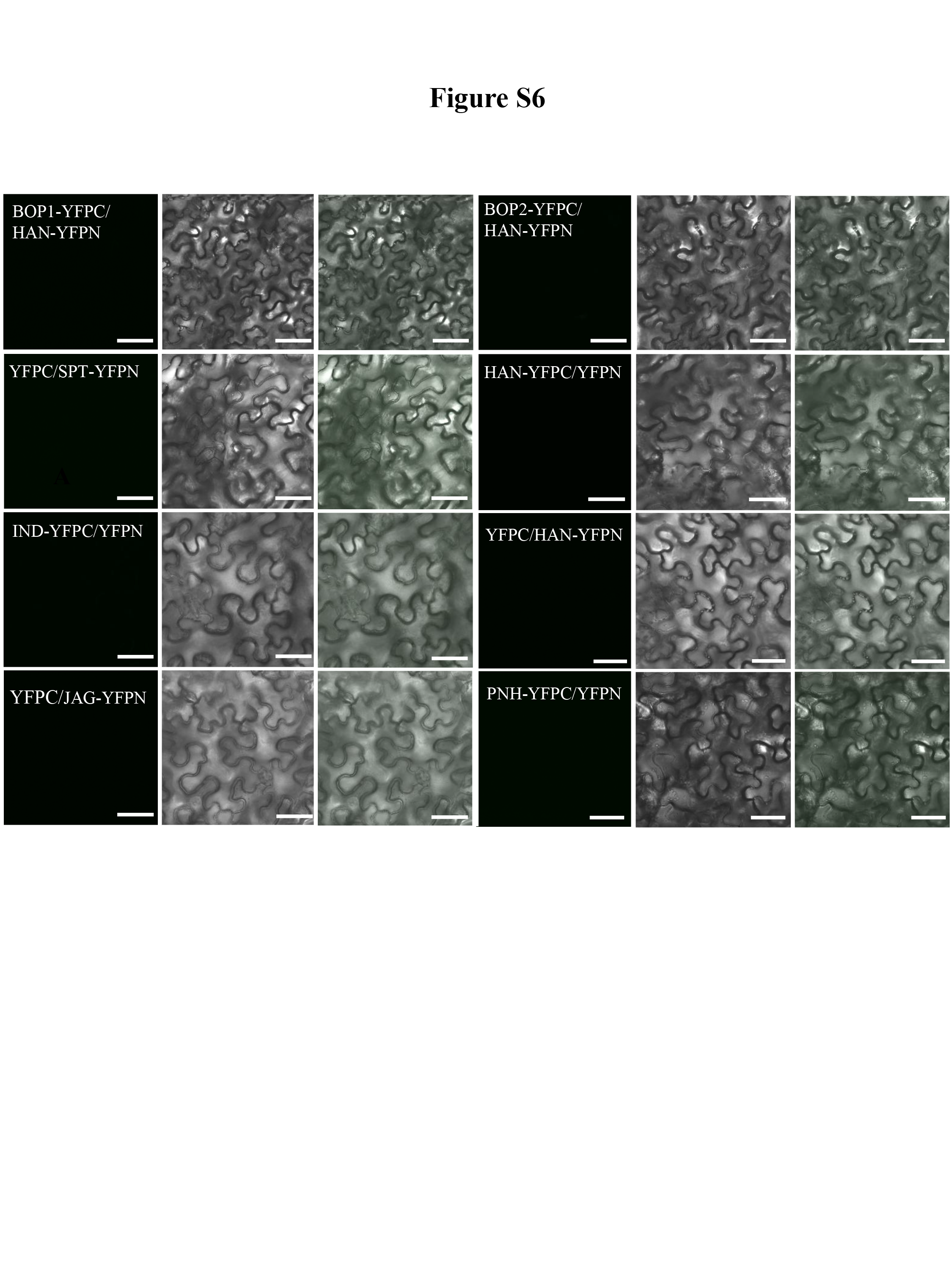

Supplement: S6 Fig — The label SPT-YFPN represents the SPT fused with N-terminus half of YFP in-frame, and similarly for other constructs. A positive interaction was shown by YFP fluorescence (green) in nuclei (left panel). Differential interference contrast images of the tobacco cells are shown in the middle panel, and the two channels merged are shown in the right panel. Bars = 50μm. (TIF) [file pgen.1005479.s006.tif]

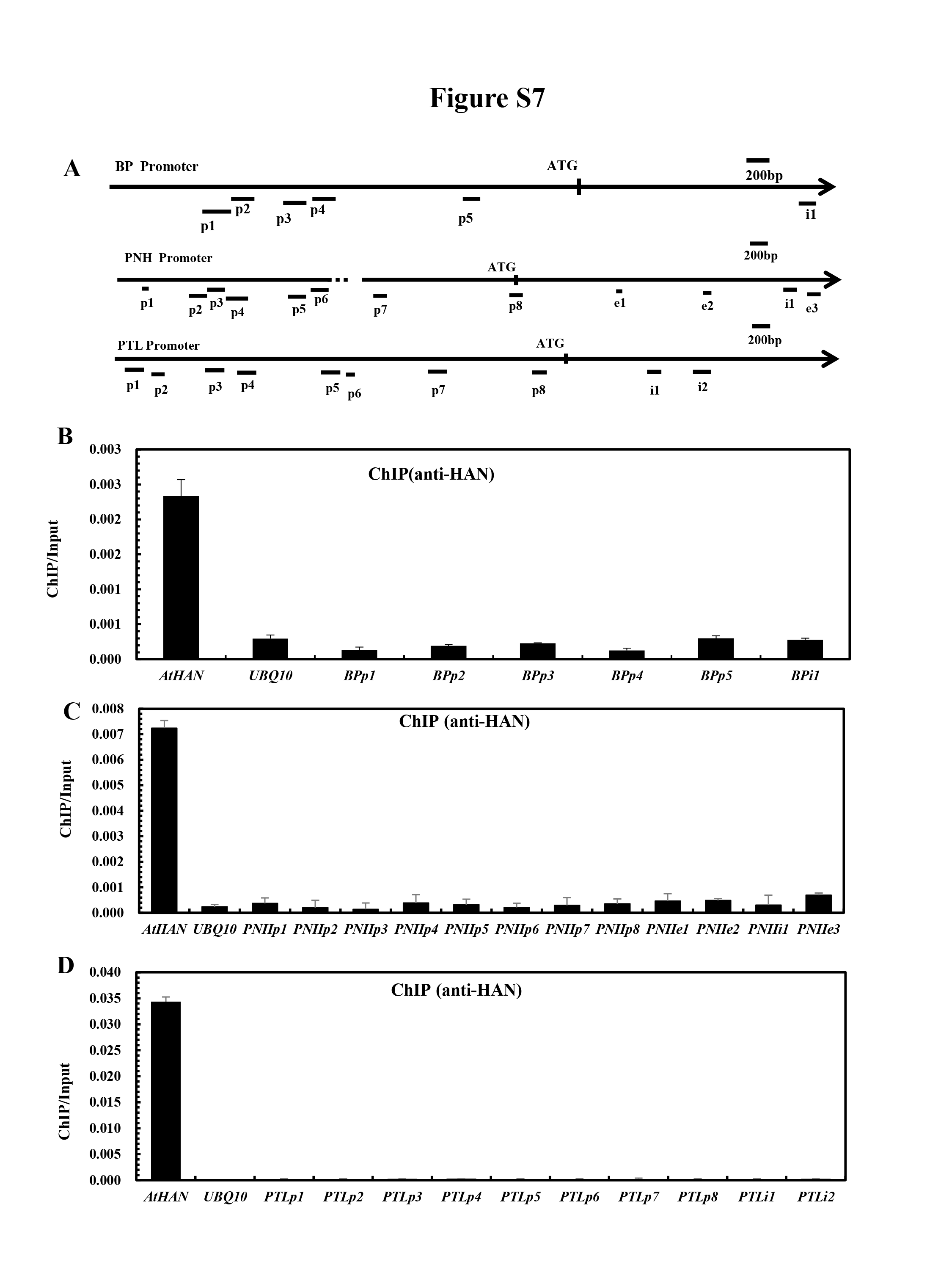

Supplement: S7 Fig — (A) Schematic diagram of the amplicons located in the BP, PNH and PTL genomic sequence used for ChIP analyses. Letter p represents promoter, i represents intron, e indicates exon, u represents UTR. (B-D) ChIP PCR assay with anti-HAN antibody showed no enrichment of amplicons from BP (B), PNH (C) and PTL (D in wild-type Ler inflorescence. The data were the average of two biological replicates. HAN and UBQ10 were used for positive and negative controls, respectively. (TIF) [file pgen.1005479.s007.tif]
