# Supplementary material for: HANABA TARANU (HAN) Bridges Meristem and Organ Primordia Boundaries through PINHEAD, JAGGED, BLADE-ON-PETIOLE2 and CYTOKININ OXIDASE 3 during Flower Development in Arabidopsis
Source: PLoS Genet. 2015 Sep 21;11(9):e1005479. doi: 10.1371/journal.pgen.1005479 (PMC4577084; doi:10.1371/journal.pgen.1005479)
Supplement: S1 Table — (DOCX) [file pgen.1005479.s008.docx]

| **Table S1. Primer information used in this study** | |
| --- | --- |
| **Primers for genotyping** | |
| *HAN-gt-F* | 5'- ACTCCTTCTACGAGGCTTTGTG-3' |
| *HAN-gt-R* | 5'- TGAGCCCACGGAGTACCATTAT-3' |
| *gHAN-2334-seq* | 5'- TACCATATCTACTTAAACCAAACTTCA-3' |
| *HAN1-gt-R* | 5'- AGAGTGAGATCACAGATTCAGGAAC-3' |
| *gHAN-3125-R* | 5'- CATCGCTCTTACACACTTCTCT-3' |
| *PTL-gt-F* | 5'- GATCAGCATCCTCAGTACGGTA-3' |
| *PTL-gt-R* | 5'- AGATGGAAGCTACGACTTAGTGTT-3' |
| *PNH-gt-F* | 5'- AACTTAATTCGACTTCTACCTTTGTAGCCATGCGGGAATT-3' |
| *PNH-gt-R* | 5'- AATACCATCTGCTGTGAAATTGTTCTCGTC-3' |
| *o8474* | 5'- ATAATAACGCTGCGGACATCTACATTTT-3' |
| *BOP1-gt-F* | 5'- TTCTAGCCAAACATCTCCCAAT-3' |
| *BOP1-gt-R* | 5'- CGTGTGTTCGTCTTTCATCCTA-3' |
| *BOP2-gt-F* | 5'- AAGAAAGCAAGACATGCATCAA-3' |
| *BOP2-gt-R* | 5'- TCTCGAGATATCACCATTGCAG-3' |
| *dspm1-RB* | 5'- CTTATTTCAGTAAGAGTGTGGGGTTTTGG-3' |
| *JAG-gt-F* | 5'- TAAGTAGTAGTCGTTGAAGTGGTTG-3' |
| *JAG-gt-R* | 5'- GTACTCTCTCTTCCAAAAAAATTTC-3' |
| **Primers for qRT-PCR** | |
| *qHAN-F* | 5'- TCTCCGCTAACAAGCCAAGT-3' |
| *qHAN-R* | 5'- GAGCCCACGGAGTACCATTA -3' |
| *qJAG-F* | 5'- ATCTCTCCAAGTCCTAACCTCCC -3' |
| *qJAG-R* | 5'- AGCGTCACCATCACGACCTTG -3' |
| *qPNH-F* | 5'-AGAATCGTAGCCGGAGGAGAA-3' |
| *qPNH-R* | 5'-GCATCTGATGATTCACAGCGAC-3' |
| *qPTL- F* | 5'-AACGAACAAGGCAATGTAGGTT-3' |
| *qPTL- R* | 5'-TGAGCCTCAAACCATAACTCTCC-3' |
| *qBP-F* | 5'-AATAGTAGCAATTATGGTCCTGG-3' |
| *qBP-R* | 5'-CTTGAGTATTGTGGATGGCTCT-3' |
| *qBOP2-F* | 5'-CGTTGAAGGTCGTTTAGTCCA -3' |
| *qBOP2-R* | 5'-TGACAGGTTGTGGTGAGTCTG-3' |
| *qBOP1-F* | 5'-TGACAAGTATGGTGGAGAAAGC-3' |
| *qBOP1-R* | 5'-GGAGAGGCATTGAAGATTTGAG-3' |
| *qCKX3-F* | 5'-CTATGAATCGCAACAAGTGGAA -3' |
| *qCKX3-R* | 5'-ACCTCGGACCAAAATGTCTAAC -3' |
| *ACTIN2-F* | 5'-CCTTCGTCTTGATCTTGCGG-3' |
| *ACTIN2-R* | 5'-AGCGATGGCTGGAACAGAAC -3' |
| **Primers for *in situ* probes** | |
| *HAN-SP6* | 5'-GATTTAGGTGACACTATAGaatGCTAAACCTGCAAACTGAACTTATCTC -3' |
| *HAN-T7* | 5'-tgTAATACGACTCACTATAGGGGTTAGACCGTCACTATCAATAAACA -3' |
| *BOP1-SP6* | 5'-GATTTAGGTGACACTATAGaatGCTATAGTCTCTCTCTCTCTTCTT -3' |
| *BOP1-T7* | 5'-tgTAATACGACTCACTATAGGGACCATAACAACAATTTAATTAGATATTG -3' |
| *BOP2-SP6* | 5'-GATTTAGGTGACACTATAGaatGCTCTTTCAGAGAGGAGGAGCAA -3' |
| *BOP2-T7* | 5'-tgTAATACGACTCACTATAGGGTAAAGATAAGATATTAATCGATGGCA -3' |
| *JAG-Sp6* | 5'- GATTTAGGTGACACTATAGaatGCTAGAGGGAGACCGAGACATTGAA -3' |
| *JAG-T7* | 5'- tgTAATACGACTCACTATAGGGCAGAGCGAGTGATGATCTTGAAA -3' |
| *PNH-SP6* | 5'-GATTTAGGTGACACTATAGaatGCTTTGTCGAAGCCATTGTCGG -3' |
| *PNH-T7* | 5'-tgTAATACGACTCACTATAGGGTATCAAACCAAGCTCGGTTTCA -3' |
| *BP-sp6* | 5'-GATTTAGGTGACACTATAGaatGCTCCTTGACGAATTCTATATACCTAGT -3' |
| *BP-T7* | 5'-tgTAATACGACTCACTATAGGGATTGTTGATTTGTTTCTGATCTAA -3' |
| *PTL-SP6* | 5'-GATTTAGGTGACACTATAGaatGCTGAGGATGGAAGCTAGGGATG -3' |
| *PTL-T7* | 5'-tgTAATACGACTCACTATAGGGGAGCCTCAAACCATAACTC -3' |
| *CKX3-SP6* | 5'- GATTTAGGTGACACTATAGaatGCTCGAAAACGGACGGTGTAGAT -3' |
| *CKX3-T7* | 5'-TGTAATACGACTCACTATAGGGCACCTCGGACCAAAATGTCT -3' |
| **Primers for yeast two-hybrid** | |
| *HAN-YTH-F* | 5'-GGAATTCCATATGATGATGCAGACTCCGTACACT -3' |
| *HAN -YTH-R* | 5'-CGGGATCCCTCTGGTAAAGTCATGGACAAGAC -3' |
| *BP-YTH-F* | 5'-CCGGAATTC ATGGAAGAATACCAGCATGACA -3' |
| *BP-YTH-R* | 5'-CGGGATCCC TTATGGACCGAGACGATAAGG -3' |
| *PTL-YTH-F* | 5'-GGAATTCCATATG ATGGATCAAGATCAGCATCCT -3' |
| *PTL-YTH-R* | 5'-CGGGATCCC TTACTGATTCTCTTCTTTACTGAGC -3' |
| *JAG-YTH-F* | 5'- CATATG ATGAGGCATGAGGAGAATTACTTAG -3' |
| *JAG-YTH-R* | 5'- GGATCCCTCAGAGCGAGTGATGATCTTGA -3' |
| *PNH-YTH-F* | 5'-TCCCCCGGGG ATGCCGATTAGGCAAATG -3' |
| *PNH-YTH-R* | 5'-CGGGATCCC TTAGCAGTAGAACATTACTCTCTTC -3' |
| *PNH-Ⅰ-F* | 5'-TCCCCCGGGGATGCCGATTAGGCAAATGAAA -3' |
| *PNH-Ⅰ-R* | 5'-CGGGATCCCCTAAGACCCTTCTTAATCTTGACGC -3' |
| *PNH-Ⅱ-F* | 5'-TCCCCCGGGGAGGAGTGAAAGTAGAGGTTACTCAC -3' |
| *PNH-Ⅱ-R* | 5'-CGGGATCCCCGTTCTCGTGATACTTAAGCC -3' |
| *PNH-Ⅲ-F* | 5'-TCCCCCGGGGGGAAAGAAAAAGATTGTCTCC -3' |
| *PNH-Ⅲ-R* | 5'-CGGGATCCCTTAGCAGTAGAACATTACTCTCT -3' |
| *PNH-Δ1-F* | 5'-TCCCCCGGGGATGCCGATTAGGCAAATGAA -3' |
| *PNH-Δ1-R* | 5'-CGGGATCCCTTTCCCGTTCTCGTGATACT -3' |
| *PNH-Δ2-F* | 5'-TCCCCCGGGGAGGAGTGAAAGTAGAGGTTACT -3' |
| *PNH-Δ2-R* | 5'-CGGGATCCCTTAGCAGTAGAACATTACTCTCTTC -3' |
| *Bop1-YTH-F* | 5'-GGAATTCATGAGCAATACTTTCGAAGAATCA -3' |
| *Bop1-YTH-R* | 5'-CGGGATCCTCTAGAAATGGTGGTGGTGGTG -3' |
| *Bop2-YTH-F* | 5'-GGAATTCATGAGCAATCTTGAAGAATCTTTGA -3' |
| *Bop2-YTH-R* | 5'-CGGGATCCTCTAGAAGTGATGTTGATGATGGTGA -3' |
| **Primers for BiFC** | |
| *IND-BiFC-F* | 5'-TGC**TCTAGA** ATGGAAAATGGTATGTATAAAAAG -3' |
| *IND-BiFC-R* | 5'-CGCGGATCC GGGTTGGGAGTTGTGGTA -3' |
| *SPT-BiFC-F* | 5'-TGCTCTAGA ATGATATCACAGAGAGAAGAAAGA -3' |
| *SPT-BiFC-R* | 5'-CGCGGATCC AGTAATTCGATCTTTTAGGTCAG -3' |
| *BP-BiFC-F* | 5'-CGCGGATCC ATGGAAGAATACCAGCATGACA -3' |
| *BP-BiFC-R* | 5'-TCCCCCGGGTGGACCGAGACGATAAGGT -3' |
| *JAG-BiFC-F* | 5'- TCTAGA ATGAGGCATGAGGAGAATTACTTAG -3' |
| *JAG-BiFC-R* | 5'- GGATCC GAGCGAGTGATGATCTTGA -3' |
| *PNH-BiFC-F* | 5'-TGCTCTAGA ATGCCGATTAGGCAAATG -3' |
| *PNH-BiFC-R* | 5'-CGCGGATCC GCAGTAGAACATTACTCTCTTCA -3' |
| *HAN-BiFC-F* | 5'-CTAGACTAGTATGATGCAGACTCCGTACACT-3' |
| *HAN-BiFC-R* | 5'-A TCCCCCCGGGTCTGGTAAAGTCATGGACAAGAC-3' |
| *BOP1-BiFC-F* | 5'-GGACTAGTATGAGCAATACTTTCGAAGAATC-3' |
| *BOP1-BiFC-R* | 5'-CGGGATCCCTAGAAATGGTGGTGGTGGT-3' |
| *BOP2-BiFC-F* | 5'-GCTCTAGAATGAGCAATCTTGAAGAATCTTT-3' |
| *BOP2-BiFC-R* | 5'-CGGGATCCCTAGAAGTGATGTTGATGATGGTG-3' |
| **Primers for ChIP-PCR** | |
| *CKX3p4-F* | 5'- GCAAATGGAATTGGAGTGTTTTT -3' |
| *CKX3p4-R* | 5'- GCCGTAAATAAACGATAAAAAGCA -3' |
| *CKX3i3-F* | 5'- CGCCTCAAAAATGAAATGGAC -3' |
| *CKX3i3-R* | 5'- TTTGAGTTTTGTCCTTACGCCA -3' |
| *JAGp9-F* | 5'- CACATGACCACCTCTTTCTTTTTAA -3' |
| *JAGp9-R* | 5'- CACAAAAGGAAGAGAAAAACGAAT -3' |
| *BOP1-F* | 5'-ACCACATAAGCACGAGCCAAG-3' |
| *BOP1-R* | 5'-AGTACCTGAGTAAGCAACGCGAG-3' |
| *BOP2-F* | 5'-CTTTGCCTCGAGCTTGTTCAA-3' |
| *BOP2-R* | 5'-TGTTATTGCTCCCTCCACTGC-3' |
| *UBQ10-F* | 5'- TCCAGGACAAGGAGGTATTCCTCCG -3' |
| *UBQ10-R* | 5'- CCACCAAAGTTTTACATGAAACGAA -3' |
| *HAN-F* | 5'-CTCATCACCACTCGACGCAG -3' |
| *HAN-R* | 5'-CCAAGAAAGGAACGGAACACC -3' |
|  |  |
|  |  |
